# Early and selective localization of tau filaments to glutamatergic subcellular domains within the human anterodorsal thalamus

**DOI:** 10.1007/s00401-024-02749-3

**Published:** 2024-06-11

**Authors:** Barbara Sárkány, Csaba Dávid, Tibor Hortobágyi, Péter Gombás, Peter Somogyi, László Acsády, Tim J. Viney

**Affiliations:** 1 Department of Pharmacology, University of Oxford, Oxford, OX1 3QT UK; 2 Lendület Laboratory of Thalamus Research, Institute of Experimental Medicine, Budapest, 1083 Hungary; 3 Department of Anatomy, Histology and Embryology, Semmelweis University, Budapest, 1094 Hungary; 4 Department of Neurology, Faculty of Medicine, University of Debrecen, Debrecen, 4032 Hungary; 5 Department of Pathology, Szt. Borbála Hospital, Tatabánya, 2800 Hungary

**Keywords:** Tau, Thalamus, vGLUT2, Alzheimer’s disease, Paired helical filaments

## Abstract

Widespread cortical accumulation of misfolded pathological tau proteins (ptau) in the form of paired helical filaments is a major hallmark of Alzheimer’s disease. Subcellular localization of ptau at various stages of disease progression is likely to be informative of the cellular mechanisms involving its spread. Here, we found that the density of ptau within several distinct rostral thalamic nuclei in post-mortem human tissue (*n* = 25 cases) increased with the disease stage, with the anterodorsal nucleus (ADn) consistently being the most affected. In the ADn, ptau-positive elements were present already in the pre-cortical (Braak 0) stage. Tau pathology preferentially affected the calretinin-expressing subpopulation of glutamatergic neurons in the ADn. At the subcellular level, we detected ptau immunoreactivity in ADn cell bodies, dendrites, and in a specialized type of presynaptic terminal that expresses vesicular glutamate transporter 2 (vGLUT2) and likely originates from the mammillary body. The ptau-containing terminals displayed signs of degeneration, including endosomal/lysosomal organelles. In contrast, corticothalamic axon terminals lacked ptau. The data demonstrate the involvement of a specific cell population in ADn at the onset of the disease. The presence of ptau in subcortical glutamatergic presynaptic terminals supports hypotheses about the transsynaptic spread of tau selectively affecting specialized axonal pathways.

**Supplementary Information:**

The online version contains supplementary material available at 10.1007/s00401-024-02749-3.

## Introduction

A major function of tau is to mediate the assembly of microtubules. The accumulation and apparent spread of pathological misfolded forms of tau (ptau) are associated with a wide range of neurodegenerative diseases including Alzheimer’s disease [9, 15, 16]. However, it remains uncertain how ptau spreads from vulnerable sites in the human brain. Since animal models might not exactly recapitulate the neuroanatomical and neurochemical characteristics of its propagation, it is important to define the cellular and subcellular compartments in the human brain that are involved in the progression of ptau. Despite the primary focus on cortical regions in Alzheimer’s research, specific subcortical regions such as the rostral thalamus, locus coeruleus (LC), and dorsal raphe nucleus (DRn) have also been shown to display selective vulnerability to tau pathology [7, 29, 33, 69, 75].

Earlier reports have shown ptau, extracellular amyloid deposits, as well as cell loss in the thalamus, with the anterodorsal thalamic nucleus (ADn) being the most affected by ptau and neurodegeneration [15, 61, 85]. The ADn, together with the anteroventral (AV) and anteromedial (AM) thalamic nuclei, comprises the anterior nuclear group, which forms one functional unit with the hippocampal formation and the mammillary bodies (the so-called Papez circuit) [2, 30, 80]. As a consequence, the pathology of the anterior nuclear group has been linked to the early cognitive biomarkers for Alzheimer’s disease (episodic memory impairments and disorientation) [3]. Indeed, lesions of the anterior nuclear group impair spatial memory in rodents [32, 50, 57], and its degeneration in humans causes memory deficits [36].

In these early studies [15, 85], it was unclear whether thalamic degeneration precedes or follows the cortical pathology. The thalamus contains different cell types [31, 41, 52], but it is still unknown whether besides nuclear (regional) specificity tau pathology also displays any cell-type specificity within this subcortical structure. Furthermore, the thalamus contains a wide range of cortical and subcortical afferents [1], and examination of synaptosomes suggests that ptau may accumulate at both pre- and postsynaptic sites [72]. To what extent ptau can be expressed in various axon terminals and whether ptau displays any specificity based on the origins of its inputs have never been investigated in the thalamus.

The apparent ‘prion-like’ spread of ptau is associated with a wide range of neurodegenerative diseases including Alzheimer’s disease [9, 15, 16, 18, 21]. Early electron microscopic studies of sporadic cases have identified ptau in argyrophilic tubules of astrocytes [10, 53] and in oligodendroglia [8] of highly devastated neuropil. Furthermore, ptau also labels tubular structures associated with swollen neurite aggregation surrounding extracellular neurofibrillary tangles in the hippocampus of progressive supranuclear palsy (PSP) cases [11]. The three-repeat and four-repeat isoforms of tau have been identified in neurons and glia of various tauopathies (Pick’s disease, PSP, corticobasal degeneration) [9]. More recently, three forms of tau (‘oligomeric’, ‘misfolded’ and ‘phosphorylated’) have been identified in pre- and postsynaptic sites in the cortex using array tomography [23]. However, high-quality, quantitative demonstration of ptau distribution at the electron microscopic level in various neuronal compartments of the thalamus has not been performed at different stages of the disease and it is exceedingly rare in other regions of the brain as well. Thus, in this study we asked at exactly what disease stages thalamic tau pathology appears and whether ptau displays any cell-type specificity. We also examined whether ptau is restricted to somatodendritic compartments of thalamocortical cells and whether in case of axonal labeling what types of axons are affected. We found that a consistent pattern of thalamic pathology appears prior to the widespread cortical pathology and is highly selective for the calretinin-containing neurons of the ADn. We also demonstrate that ptau selectively accumulates not only in thalamocortical cells, but also in their major subcortical afferents arising from the mammillary body, supporting theories about the trans-neuronal spread of the disease.

## Materials and methods

Details of antibodies, reagents and resources can be found in Tables S1 and S2.

### Human samples

Human brain samples from *n* = 26 cases were obtained from the Department of Pathology, Szt. Borbála Hospital, Tatabánya, Hungary via the Human Brain Research Laboratory (HBL, Institute of Experimental Medicine, Hungary), the MRC London Neurodegenerative Diseases Brain Bank (KCL, King’s College London, UK), the Queen Square Brain Bank for Neurological Disorders (QSBB, UCL, London, UK), and the Oxford Brain Bank (OBB, Oxford, UK). The reported Braak tau stage [14, 16] for each case was based on standardized examination of the severity and extent of ptau in the cerebral cortex (AT8 immunoreactivity). Braak stage 0 is defined by a lack of cortical tau pathology and no detected/reported cognitive impairment (‘control’ cases). At stage I, ptau is observed in the entorhinal cortex (EC), followed by the hippocampus at stage II. By stage III, ptau is located in other areas of the medial temporal lobe and is associated with amyloid-ß plaques and mild cognitive impairment. Alzheimer’s disease is associated with the later stages, with ptau having spread beyond the medial temporal lobe. Psychiatric conditions for the HBL cases (Table 1) were previously assessed at the Department of Psychiatry, Szt. Borbála Hospital, Tatabánya, Hungary.

**Table 1:** List of cases

Case	Group	Braak tau stage	Clinical diagnosis (neuro/psych)	Age	Sex	Thal (Aβ) phase	CERAD	NIA-AA ABC score for AD	AD neuropathologic change NIA-AA 2012	Primary pathological diagnosis	Further pathological diagnosis	APOE	PMD (h)	Cause of death	Major comorbidities	Sections	Source
1	Early	0	WNL	86	m	0	0	A0B0C0	Not AD	WNL		E3/E3	6	AMI		FFPE	KCL
3	Early	0	Schizophrenia	72	m	0	0	A0B0C0	Not AD	WNL	Mild SVD	NA	2.5	Cardiac arrest	IHD, COPD	PFFF	HBL
4	Early	0	Schizophrenia	57	f	0	0	A0B0C0	Not AD	WNL		NA	3	Respiratory arrest	CHF, COPD	PFFF	HBL
5	Early	0	Schizophrenia	71	f	4	NA	A3B0C0	Low	WNL		NA	4	Heart failure	IHD, HT, art.scler., obesity	PFFF	HBL
22	Early	0	WNL	61	f	0	0	A0B0C0	Not AD	WNL		NA	3	AMI	Art.scler., obesity, HT, NIDDM	PFFF	HBL
24	Early	0	WNL	61	f	0	0	A0B0C0	Not AD	WNL	HTE	NA	5.05	Cardiogenic shock	IHD	PFFF	HBL
20	Early	I	WNL	59	f	3	1	A2B1C1	Low	ADNC		NA	26.3	AMI	Art.scler	FFIF	OBB
6	Early	I	WNL	65	m	2	0	A1B1C0	Low	PART	Mild SVD	NA	53	Bronchopneumonia	Art.scler	FFPE	QSBB
7	Early	I	WNL	71	m	1	1	A1B1C1	Low	ADNC	Mild CAA	E3/E3	24	Disseminated cancer		FFPE	KCL
8	Early	I	WNL	74	m	NA	0	AxB1C0	Not AD	WNL		E3/E3	42	Pancreatic cancer		FFPE	KCL
9	Early	I	WNL	81	m	1	0	A1B1C0	Low	PART	Mild SVD	NA	2.5	Bronchopneumonia	Art.scler., IHD	PFFF	HBL
2	Early	II	WNL	72	f	2	1	A1B1C1	Low	ADNC	Mild CAA	NA	36	Disseminated cancer		FFPE	QSBB
23	Early	II	WNL	87	f	1	0	A1B1C0	Low	WNL		NA	3	NA		PFFF	HBL
25	Early	II	WNL	75	f	0	0	A0B1C0	Not AD	PART	Mild SVD	NA	4.5	AMI	Art.scler., COPD, obesity	PFFF	HBL
26	Early	II	WNL	85	f	1	0	A1B1C0	Low	PART	Mild SVD, CAA	NA	3	CHF	Acute peritonitis, COPD	PFFF	HBL
10	Middle	III	WNL	86	m	NA	NA	AxB1Cx	Not AD	PART		E3/E3	52	CHF		FFPE	KCL
11	Middle	III	NA	80	f	2	1	A1B2C1	Low	ADNC	Moderate SVD	NA	3	Ileus	IHD, HT	PFFF	HBL
12	Middle	III	NA	82	f	2	3	A1B2C3	Intermediate	ADNC		NA	2	AMI	IHD, HT	PFFF	HBL
13	Middle	III	Schizophrenia	84	m	1	0	A1B2C0	Low	ADNC	Moderate SVD	NA	3	Respiratory arrest	COPD	PFFF	HBL
21	Middle	III	WNL	77	f	4	1	A3B2C1	Intermediate	ADNC		E3/E4	69	NA	Chr. arthritis, HT	FFIP	OBB
14	Middle	IV	NA	89	f	0	0	A0B2C0	Not AD	AgD		NA	2.5	NA		PFFF	HBL
15	Late	V	Circumscribed brain atrophy	78	m	5	3	A3B3C3	Definite	ADNC	Mild SVD; Limbic LBD	NA	5	Rectal cancer		FFPE	QSBB
16	Late	V	AD	71	m	NA	3	AxB3C3	High	ADNC	Mild CAA	E3/E4	5	Hemorrhagic shock		FFPE	KCL
17	Late	VI	NA	81	f	4	3	A3B3C3	Definite	ADNC	Moderate SVD; LATE;	NA	2	NA		PFFF	HBL
18	Late	VI	AD	72	m	NA	3	AxB3C3	Intermediate ≤	ADNC	Severe CAA	E3/E3	5	NA		FFPE	KCL
19^a^	Late	VI	NA	76	f	NA	3	AxB3C3	Intermediate ≤	ADNC		NA	2.2	Heart failure	Art.scler., IHD, HT	PFFF	HBL

CERAD neuritic plaque score: none (0), sparse (1), moderate (2), frequent (3)

*WNL* within normal limits, *NA* not available, *AD* Alzheimer’s disease, *CERAD* Consortium to Establish a Registry for Alzheimer’s disease, *NIA-AA* National Institute on Aging and Alzheimer’s Association, x unknown, *PART* primary age-related tauopathy, *ADNC* Alzheimer’s disease neuropathologic change, *SVD* small vessel disease, *HTE* hypertensive encephalopathy, *CAA* cerebral amyloid angiopathy, *AgD* argyrophilic grain disease, *LBD* Lewy body disease, *LATE* limbic-predominant age-related TDP-43 encephalopathy neuropathologic change, *PMD* post-mortem delay time, *AMI* acute myocardial infarction, *CHF* congestive heart failure, *IHD* chronic ischemic heart disease, *COPD* chronic obstructive pulmonary disease, *HT* hypertension, *NIDDM* non-insulin-dependent diabetes mellitus, *Art.scler* arteriosclerosis, *Chr.* chronic, *FFPE* formalin-fixed paraffin-embedded (10-µm-thick sections), *PFFF* perfusion-fixed free-floating (50-µm-thick free-floating sections), *FFIF* flash-frozen immersion fixed (50-µm-thick free-floating sections), *KCL* London Neurodegenerative Diseases Brain Bank, *HBL* Human Brain Research Laboratory, *OBB* Oxford Brain Bank, *QSBB* Queens Square Brain Bank

^a^Only cortical sections were available

Perfusion-fixed free-floating (PFFF) sections: Perfusion-fixed tissue blocks from the HBL (Table 1) were received as previously described [33] and are from the same collection of cases reported by Gilvesy et al. [33], but with different numbering. Briefly, brains were removed 2–4 h post-mortem and vertebral arteries and internal carotid were cannulated. Perfusion was carried out using physiological saline containing 0.33% heparin (1.5 L for 30 min), followed by Zamboni fixative containing 4% paraformaldehyde and ~0.2% w/v picric acid in 0.1 M phosphate buffer (PB), pH = 7.4 (4 L for 2 h). Tissue blocks were removed and post-fixed overnight in the same solution then washed and stored in 0.1 M PB with 0.05% sodium azide (PB-Az). Experiments were performed in compliance with the 1964 Declaration of Helsinki, approved by the Regional Committee of Science and Research Ethics of Scientific Council of Health (ETT TUKEB 31443/2011/EKU and ETT TUKEB 15032/2019/EKU). Blocks were serially sectioned into 50-µm-thick coronal sections using a Leica VTS-1000 Vibratome (Leica Microsystems, Wetzlar, Germany). Free-floating sections were incubated in 20% sucrose for cryoprotection then subjected to freeze–thaw over liquid nitrogen. Other sections were not subjected to freeze–thaw. Next, the PFFF sections were incubated in 1% hydrogen peroxide to reduce endogenous peroxidase activity. Sections were washed several times in 0.1 M PB and then stored in PB-Az at 4 °C.

Flash-frozen immersion fixed (FFIF) sections: Flash-frozen samples were obtained from the OBB (Table 1) under project OBB 606 (ethics approval 15/SC/0639). Briefly, the rostral thalamus was isolated from the relevant slab over dry ice and immediately immersed in fresh 4% paraformaldehyde, ~0.2% w/v picric acid, and 0.05% glutaraldehyde in 0.1 M PB. Samples were microwaved for up to 30 s for rapid thawing. Samples were post-fixed in fixative lacking the glutaraldehyde overnight, then washed several times in 0.1 PB and processed as above for PFFF sections.

Formalin-fixed paraffin-embedded (FFPE) sections: Tissue blocks were obtained from KCL (Table 1) under Tissue Bank ethics approval (18/WA/0206). A microtome (Reichert-Jung, 2035) was used to prepare 5–10 μm thick sections (up to ~300 sections per block) and transferred onto slides (Superfrost Plus) in 37 °C water. A series of 10-μm-thick tissue sections were also obtained from QSBB (Table 1) under Tissue Bank ethics approval 23/LO/0044. In preparation for immunohistochemical tests, sections were first deparaffinized in xylene (100%) and rehydrated in a descending ethanol series (100%, 95%, 70%, 50%). Masking of epitopes caused by fixation was reversed using antigen retrieval by incubating sections in 10 mM sodium citrate buffer at pH 6 at 90 °C for 30 min.

### Fluorescent immunohistochemistry

PFFF sections were blocked for 45 min in 4% bovine serum albumin (BSA; Sigma), and FFIF and FFPE sections were blocked in 10% or 20% normal horse serum (Vector Lab), followed by a 3-day incubation in primary antibody solution (mouse anti-AT8 diluted 1:5000, rabbit anti-calretinin (CR) diluted 1:2000; Table S1) in 0.1 PB at 4 °C. Sections were washed three times in 0.1 M PB and then incubated in secondary antibody solution (anti-mouse Alexa Fluor 488 1:1000, anti-rabbit Cy3 1:400) in 0.1 M PB for 1 h at room temperature (RT) or overnight at 4 °C. Finally, sections were mounted in Vectashield. Every experiment included controls for the method by leaving out the primary antibodies from the full protocol.

### Brightfield immunohistochemistry

For light microscopic visualization, sections were blocked in 4% normal goat serum (NGS) or 4% BSA in 0.1 M PB. This was followed by incubation with primary antibodies (1–3 days): mouse anti-AT8 1:5000, rabbit anti-calretinin 1:1000, guinea pig anti-vGLUT2 1:500, mouse anti-vGLUT2 1:8000. We tested two other antibodies that recognize different epitopes of ptau (mouse anti-PHF1 1:1000, mouse anti-CP13 1:1000; Table S1) and observed similar distributions for ptau to that of AT8. After washing in 0.1 M PB, sections were reacted with biotinylated secondary antibodies diluted in 0.1 M PB and incubated overnight at 4 °C. Subsequently, for experiments that included anti-vGLUT2 tests, sections were incubated with 1:100 avidin + biotin-HRP (horseradish peroxidase) complex (Vector Labs) in 0.1 M PB at 4 °C. The vGLUT2 immunoreaction was enhanced with tyramide signal amplification (1:50; Akoya Biosciences). Next, sections were processed using 0.5 mg/ml diaminobenzidine (DAB; Sigma-Aldrich) as chromogen, 2% nickel ammonium sulfate, and 0.4% ammonium chloride in 0.1 M PB. Subsequently, hydrogen peroxide was added to a final concentration of 0.002% w/v to initiate DAB polymerization. After 12–20 min (depending on immunolabeling intensity), reactions were stopped by washing 3 × 10 min in 0.1 M PB. Free-floating sections were fixed onto glass sides using chrome alum gelatin. Next, sections were incubated in xylene for 5 min and mounted in DPX (Merck).

### Initial electron microscopic assessment of brain sections

To assess the subcellular structure of thalamic sections without any immunoreactions, sections were post-fixed in glutaraldehyde fixative (2.5% glutaraldehyde, ~0.2% w/v picric acid, 4% paraformaldehyde in 0.1 M PB) for 1–2 h. Subsequently, sections were treated with 0.5% OsO_4_ in 0.1 M PB washed in 0.1 M PB and in distilled water. Next, sections were incubated in 50% and 70% ethanol, then with 1% uranyl acetate dissolved in 70% ethanol. Dehydration of sections was continued in an ascending alcohol series 70%, 90%, 95%, 100%) followed by acetonitrile. Finally, sections were embedded in epoxy resin (Durpucan AMC, Fluka, Sigma-Aldrich).

### Single- and double-labeling pre-embedding immunohistochemistry for combined light and electron microscopy

Free-floating sections (Table 1) were washed 3× in 0.9% NaCl buffered with 50 mM Tris (pH 7.4; TBS) and then blocked in 4% NGS in TBS for 45 min. Next, sections were incubated with primary antibodies in TBS for 3 days at 4 °C. The following conditions were used:

1. One primary antibody (AT8) with immunogold labeling visualized by silver intensification.
2. One primary antibody (vGLUT2) with peroxidase reaction.
3. Two primary antibodies, followed by a silver-intensified immunogold reaction (AT8) and a peroxidase reaction (vGLUT2).
4. Method control: no primary antibody; immunogold and biotinylated secondary antibodies, followed by silver intensification and DAB treatment.

Sections were subsequently rinsed 3× in TBS and blocked for 30 min at RT in 0.1% cold water fish skin gelatin (CWFS) solution containing 0.8% NGS diluted in TBS to reduce the non-specific binding of secondary antibodies. Sections were incubated overnight at 4 °C in CWFS solution containing biotinylated secondary antibody and/or immunogold-conjugated secondary antibodies (1:200 donkey anti-mouse ultra-small immunogold (Aurion, 100.322). Sections were washed 3× in TBS and 1× in 0.1 M PB followed by incubation in 2% GA diluted in 0.1 M PB to fix the secondary antibody conjugated to immunogold particles.

After repeated washes in 0.1 M PB and TBS, sections were incubated overnight at 4 °C in avidin + biotin-HRP complex diluted in TBS. Tissues were treated with enhancement conditioning solution (ECS; Aurion) diluted 1:10 in distilled water for 3 × 5 min. To visualize immunogold particles, sections were incubated in silver enhancement solution (SE-LM, Aurion) for 20 min at 20 °C in the dark and then washed in ECS. Following 2 × 2 min washes in distilled water and 2 × 10 min washes in 0.1 M PB, peroxidase was visualized using DAB (0.5 mg/ml) as chromogen developed with 0.01% H_2_O_2_. Subsequently, sections were washed in distilled water and treated with 0.5% OsO_4_ in 0.1 M PB for 20 min on ice in the dark. To enhance contrast, sections were incubated in 1% uranyl acetate diluted in distilled water or diluted in 70% ethanol after incubation in 50% ethanol for 25 min on ice. Next, the dehydration of sections was carried out in an ascending alcohol series (50%, 70%, 90%, 95%, 100%), followed by acetonitrile; then sections were embedded in epoxy resin. After overnight incubation at RT, they were transferred onto glass slides. To polymerize epoxy resin, sections were incubated at 55 °C for 2 days. Selected regions of the thalamus were cut out and re-embedded in epoxy blocks. Series of 50–70 nm-thick sections were cut with an ultramicrotome (Leica Ultracut UTC) and collected onto Pioloform-coated single slot copper grids. Some sections were counterstained with lead citrate to increase contrast.

Specimens were studied on three microscopes: a Jeol 1010 transmission electron microscope equipped with a digital GATAN Orius camera, at the Department of Physiology, Anatomy and Genetics, Oxford University; a JEOL 1400 transmission electron microscope equipped with a Rio digital camera, at the Sir William Dunn School of Pathology, Oxford University; and a Hitachi 7100 electron microscope with a Veleta CCD camera (Olympus Soft Imaging Solutions, Germany), at the Institute of Experimental Medicine, Budapest, Hungary. Signals were observed within the same locations and also in different structures, indicating that the experiment did not produce false-positive double-labeling results.

### Data collection

Fluorescence immunohistochemistry for CR colocalization: Tissues were collected from five cases that had at least ten ptau+ cells in the ADn (Cases 1, 12, 13, 14, 17); five cases were excluded (Cases 3, 4, 5, 9, 11). Three sections/case were assessed, reporting average values.

#### Light microscopy

We assessed all 26 cases with light microscopy (Table 1), independent of Braak staging. We examined and scored combinations of the thalamus, cortex, hypothalamus, and brainstem (Table 2; Table S3). For each case, we assessed an average of three thalamic sections, three hippocampal/entorhinal sections, six cingulate gyrus sections (Brodmann areas (BA) 23, 30, 29, 26), four sections containing the mammillary bodies, and five sections of the midbrain. We calculated the average scores across different sections. Then, for cases categorized under the same Braak stage, we report the median score (Table S3). For cases that lacked sections for light microscopy, fluorescence immunohistochemistry was used to assess the distribution of ptau.

**Table 2:** Intensity scores for ptau in subcortical brain regions

Case	Group	Braak stage	ADn	AV	MD	PVT	RE	LD	TRN	MMB	LMB	TM	LC	DRn	Evaluated sections
1	Early	0	++ (2/2)	− (2/2)	− (3/3)	− (2/3), + (1/3)	− (1/1)	− (2/2)	− (3/3)	NA	NA	NA	i (1/1)	NA	FFPE
3	Early	0	− (2/2)	− (2/2)	− (2/2)	− (2/2)	NA	NA	i (1/2), − (1/2)	NA	NA	NA	NA	NA	PFFF
4	Early	0	+ (3/3)	− (3/3)	− (3/3)	i (1/2), − (1/2)	i (3/3)	NA	i (3/3)	NA	NA	NA	NA	NA	PFFF
5	Early	0	+ (2/3), − (1/3)	− (3/3)	− (3/3)	i (3/3)	− (1/1)	NA	i (3/3)	NA	NA	NA	NA	NA	PFFF
22	Early	0	− (1/1)	− (1/1)	− (1/1)	− (1/1)	NA	NA	i (1/1)	NA	NA	NA	+ (8/8)	NA	PFFF
24	Early	0	+ (2/2)	− (1/1)	− (1/1)	− (1/1)	NA	NA	− (1/1)	− (6/6)	− (5/5)	− (6/6)	NA	NA	PFFF
20*	Early	I	+ (2/2)	− (2/2)	NA	NA	NA	NA	NA	− (1/1)	− (1/1)	NA	NA	NA	FFIF
6	Early	I	NA	− (3/3)	NA	− (3/3)	− (2/2)	NA	NA	− (3/3)	− (3/3)	NA	i (1/1)	NA	FFPE
7	Early	I	+ (2/2)	− (3/3)	− (3/3)	+ (2/3), − (1/3)	− (2/2)	NA	− (3/3)	NA	NA	NA	− (1/1)	NA	FFPE
8	Early	I	NA	NA	− (3/3)	NA	NA	− (3/3)	NA	NA	NA	NA	i (1/1)	NA	FFPE
9*	Early	I	i (1/1)	− (1/1)	− (1/1)	− (1/1)	NA	NA	NA	NA	NA	NA	+ (7/8)+ + (1/8)	NA	PFFF
2	Early	II	NA	− (3/3)	NA	− (3/3)	− (1/1)	− (3/3)	− (3/3)	− (2/2)	NA	− (3/3)	NA	NA	FFPE
23	Early	II	++ (5/5)	− (3/3)	− (3/3)	i (2/3), − (1/3)	i (3/3)	NA	i (3/3)	++ (2/2)	+ (1/1)	++ (2/2)	+ (7/7)	NA	PFFF
25	Early	II	++ (1/2), + (1/2)	− (2/2)	− (2/2)	NA	NA	NA	i (2/2)	NA	NA	NA	NA	NA	PFFF
26	Early	II	+ (2/3), ++ (1/3)	− (3/3)	− (1/1)	− (3/3)	i (1/1)	NA	i (1/1)	NA	NA	NA	i (3/4), + (1/4)	++ (4/4)	PFFF
10	Middle	III	+ (1/1)	− (1/1)	− (3/3)	− (2/3), + (1/3)	− (1/1)	− (3/3)	− (3/3)	NA	NA	NA	NA	NA	FFPE
11	Middle	III	++ (3/3)	− (2/3), + (1/3)	− (2/2)	i (2/2)	+ (1/1)	NA	+ (3/3)	NA	NA	NA	++ (6/6)	NA	PFFF
12	Middle	III	++ (3/3)	− (3/3)	− (3/3)	+ (3/3)	+ (2/2)	+ (3/3)	+ (3/3)	NA	NA	NA	++ (5/5)	NA	PFFF
13	Middle	III	++ (3/3)	+ (2/3), − (1/3)	+ (1/2), − (1/2)	++ (2/2)	+ (3/3)	NA	+ (3/3)	NA	NA	NA	NA	NA	PFFF
21	Middle	III	++ (2/2)	− (2/2)	NA	NA	+ (1/1)	NA	++ (3/3)	NA	NA	NA	NA	NA	FFIF
14	Middle	IV	++ (2/3), +++ (1/3)	+ (3/3)	− (1/1)	++ (3/3)	NA	NA	i (3/3)	+++ (6/6)	+++ (3/3)	+++ (3/3)	++ (4/4)	NA	PFFF
15	Late	V	+++ (3/3)	++ (3/3)	NA	++ (3/3)	NA	NA	++ (2/2)	++ (3/3)	NA	++ (3/3)	++ (1/1)	++ (1/1)	FFPE
16	Late	V	+++ (1/1)	NA	+ (3/3)	+++ (2/3), ++ (1/3)	++ (1/1)	+++ (2/2)	++ (3/3)	NA	NA	na	NA	NA	FFPE
17	Late	VI	+++ (3/3)	++ (3/3)	+ (3/3)	++ (3/3)	++ (3/3)	na	++ (3/3)	++ (7/7)	+++ (6/6)	+++ (5/5)	+++ (8/8)	NA	PFFF
18	Late	VI	NA	− (1/1)	++ (1/2), + (1/2)	++ + (1/2), ++ (1/2)	++ (1/1)	++ (1/1)	++ (2/2)	NA	NA	NA	NA	NA	FFPE
19	Late	VI	NA	NA	NA	NA	NA	NA	NA	NA	NA	NA	NA	NA	PFFF

The distribution of ptau in each subcortical region was defined by the following scores per case: −, lacking detectable ptau; *i*, trace inclusions; +, sparse/mild; ++, moderate; +++, dense. Numbers in parentheses show the number of brain sections evaluated

*NA* not available, *FFPE* formalin-fixed paraffin-embedded (10 µm thick), *PFFF* perfusion-fixed free-floating (50 µm thick), *FFIF* flash-frozen immersion-fixed (50 µm thick)

^*^ Immunofluorescence sections were used to evaluate the thalamus

#### Electron microscopy

Samples were selected from 4/13 tested cases that had sufficient quality for analysis. Data were collected from one thalamus section/case, from which two areas were assessed. Analysis was performed by two independent individuals (B.S. and P.S.) per case; one person was blind to the Braak stages. Only synaptic structures were quantified. Electron opaque (‘dark’) boutons were excluded from the analysis due to the likely loss of antigenicity. Dark bouton frequency was as follows: Case 12, 0% (0/104), Case 25, 2.8% (*n* = 3/106), Case 4, 28.57% (*n* = 20/70); Case 17, 29.13% (*n* = 30/103). Tissue quality prevented an unbiased synapse density quantification, because of the large spaces among cellular profiles; therefore, random sampling of synapses was carried out. The frequency was influenced by the size of the boutons and dendrites. Structures were followed through 4–5 serial sections (∼4.7 sections/structure). The primary magnification was 1500–3000×, then the structures were imaged and identified at 1500–6000× magnification. Background labeling for AT8 was very low. Immunopositivity of a cellular profile for AT8 was defined by the presence of at least one silver–gold particle (∼12.1 particles/structure on average). Bouton area was measured across three serial electron micrographs and the average was calculated. Boutons without distinct membranes were not included in the analysis.

### Data analysis

The ADn was defined by the high density of CR+ cells between the MD and AV. The AV was defined by a lack of CR+ cells, located in the dorsal part of the thalamus. The nucleus reuniens could not be accurately delineated with CR; therefore, we defined the reuniens nuclear complex (RE) as the area around the third ventricle. The PVT was defined as the dorso-ventral band of CR+ cells adjacent to the midline. The MD was defined as a large nucleus predominantly lacking CR+ neurons lateral to the PVT. The TRN was identified by its net-like structure containing CR+ cells. Imaged sections were analyzed in QuPath and Python.

#### Pixel classification

To delineate thalamic nuclei in digitized AT8-immunolabeled sections, adjacent CR-immunoreacted sections were aligned with the TrakEM2 plugin in Fiji. Outlines of each thalamic nucleus were defined in the CR-immunolabeled section and imported as regions of interest (ROI) into the corresponding digitized AT8-immunolabeled section, with manual alignment required for some ROIs (Figure S1).

For quantitative analysis, ptau coverage per thalamic nucleus was detected in QuPath using an artificial neural network (ANN_MLP) pixel classifier. Only perfusion-fixed sections were assessed, as FFPE sections lacked sufficient quality. Neuronal cell bodies were counted in QuPath by manually selecting every cell to generate counts. Every case with thalamic sections was assessed (Table S4).

For ptau intensity scoring, the distribution of ptau in each area was defined by the following scores: 0, lacking detectable ptau; 0.5 (i), containing trace inclusions; 1 (+), sparse/mild; 2 (++), moderate; 3 (+++), dense (Tables 1, 2; Table S3). Both PFFF and FFPE sections were assessed. For cortical sections, scoring was carried out blind to the case/Braak stage.

### Imaging

Glass slides were digitized using a Pannoramic MIDI II scanner (3DHISTECH; Budapest, Hungary) with a Plan-Apochromat objective lens (20× magnification, NA 0.8, lateral resolution 0.346 × 0.325 μm/pixel) and pco.edge 4.2 4 MP camera. For transmitted color brightfield images, three focus levels were applied. Representative images were captured in CaseViewer. For confocal microscopy, an LSM 710 was used with Plan-Apochromat 40×/1.4, 63×/1.4, and 100×/1.46 objectives (ZEN 2008 5.0 or ZEN Black 14.0 software). Laser lines (solid-state 405 nm, argon 488 nm, HeNe 543 nm and HeNe 633 nm) were configured with the appropriate beamsplitters. The pinhole was set to ~1 Airy Unit for each channel. Brightfield images were acquired with a Zeiss AX10 microscope using an AxioCam HRc camera (63×/1.4 objective). For representative confocal images, maximum intensity projections were used for z-projections.

### Data and materials availability

Data, code, and materials used in the analysis are available on request. Human brain tissue is governed by material transfer agreements with the Brain Banks and the Human Brain Research Laboratory.

## Results

### The anterodorsal thalamic nucleus shows early and consistent vulnerability to tau pathology

**Fig. 1:**
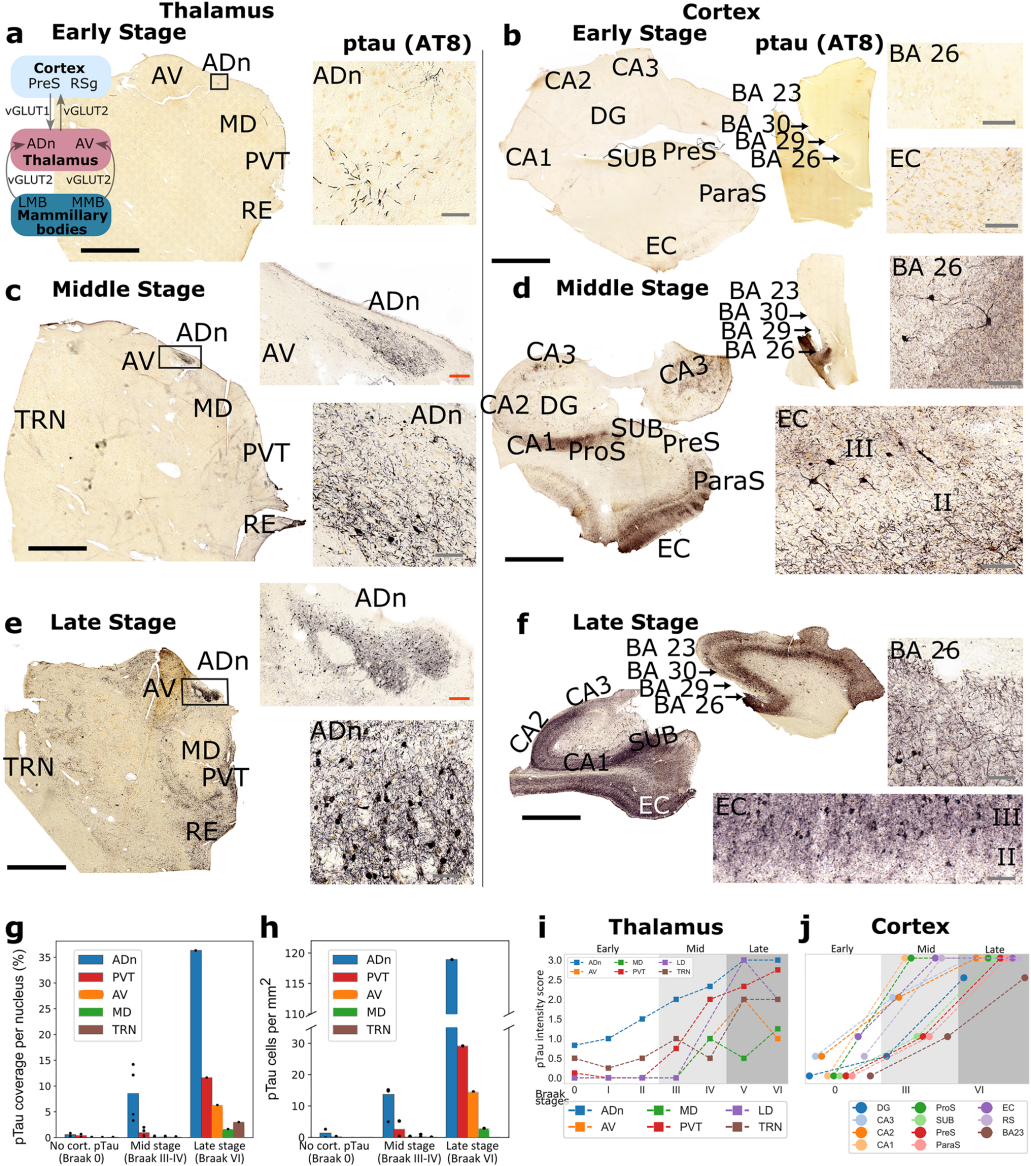
Progression of tau pathology in the rostral thalamus and the cerebral cortex. **a**–**f** Brightfield images of ptau (AT8 immunoreactivity; HRP-based diaminobenzidine (DAB) end product) in 50-µm-thick sections of rostral thalamus, hippocampal formation, parahippocampal gyrus, and cingulate gyrus. **a** Braak stage 0, Case 5. Note ptau+ dendrites in the ADn (right). Inset, schematic of neural circuit. **b** Braak stage 0, Case 5 (hippocampal formation) and Case 3 (cingulate gyrus). **c** Braak stage III, Case 12. **d** Braak stage III, Case 11 (hippocampal formation) and Case 12 (cingulate gyrus). **e** Braak stage VI, Case 17. **f** Braak stage VI, Case 19 (hippocampal formation) and Case 17 (cingulate gyrus). Quantification of ptau coverage (**g**) and ptau+ cells (**h**) for early (*n* = 2), middle (*n* = 3), and late (*n* = 1) stage cases. **i**,**j** Scoring of ptau intensity across different thalamic nuclei (*n* = 26 cases) and cortical areas (*n* = 8 cases). Scores: undetectable ptau (0), trace inclusions (0.5), sparse (1), moderate (2), dense (3). Median values are shown (see Table S4 for individual datapoints). ADn, anterodorsal nucleus; AV, anteroventral nucleus; MD, mediodorsal thalamic nucleus; PVT, paraventricular thalamic nucleus; RE, reuniens nuclear complex; TRN, reticular thalamic nucleus; DG, dentate gyrus; ProS, prosubiculum; PrS, presubiculum; ParaS, parasubiculum; EC, entorhinal cortex; BA, Brodmann area; RS, retrosplenial area (BA30, dysgranular retrosplenial cortex; BA29 and BA26, granular retrosplenial cortex); LMB, lateral mammillary nucleus; MMB, medial mammillary nucleus. Scale bars: black, 4 mm; red, 250 µm; gray, 80 µm

**Fig. 2:**
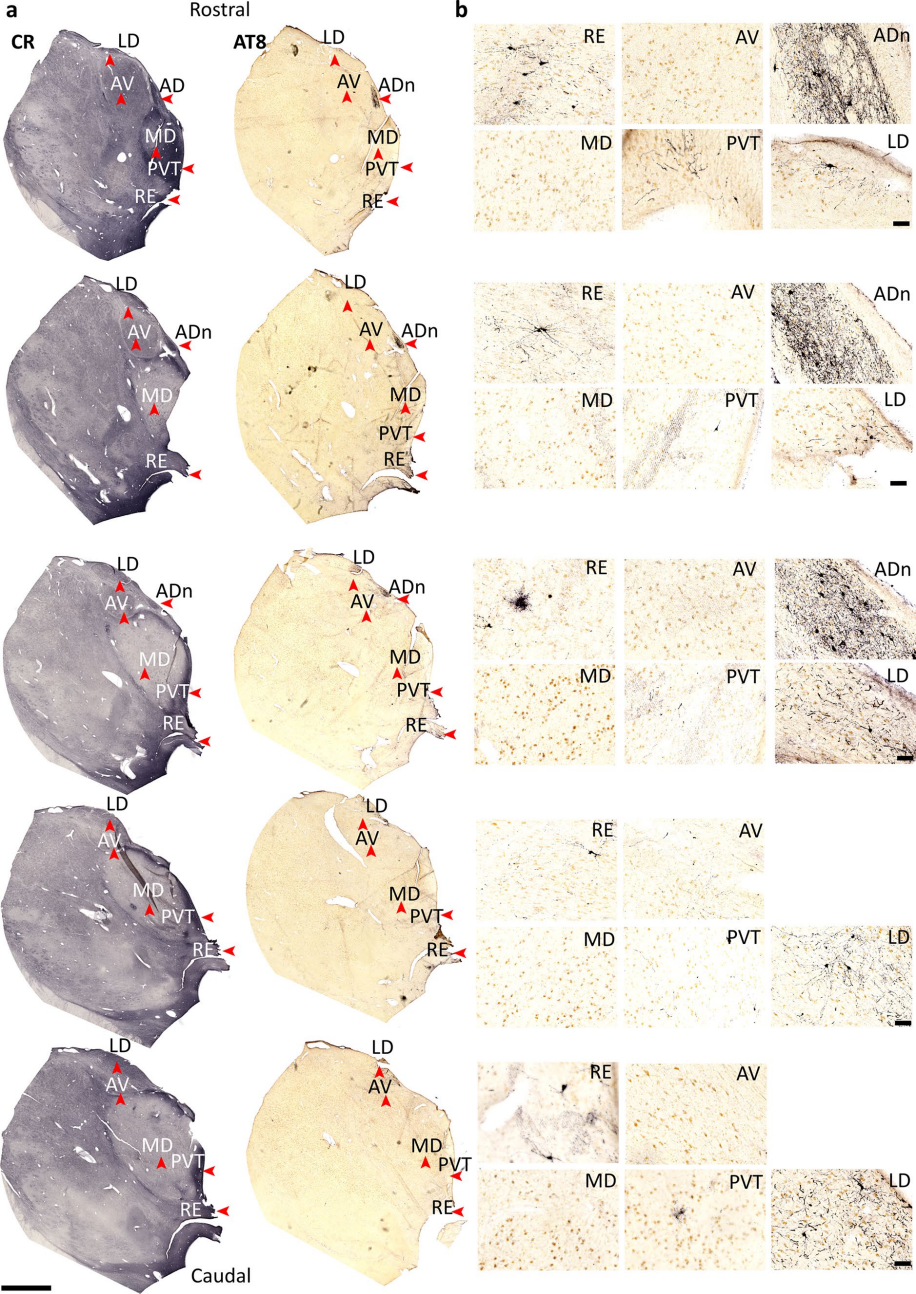
Serial sections of rostral thalamus for Braak stage III. **a**,**b** Serial sections showing the distribution of CR (left) and ptau (AT8, right) at different rostral–caudal levels by DAB–HRP immunoreaction (nickel intensified, gray/black). Note variable ptau immunoreactivity in the laterodorsal nucleus (LD) across the rostro-caudal axis. Sections are 100 µm apart (Case 12, Braak stage III). Scale bars: 4 mm (**a**), 100 µm (**b**)

**Fig. 3:**
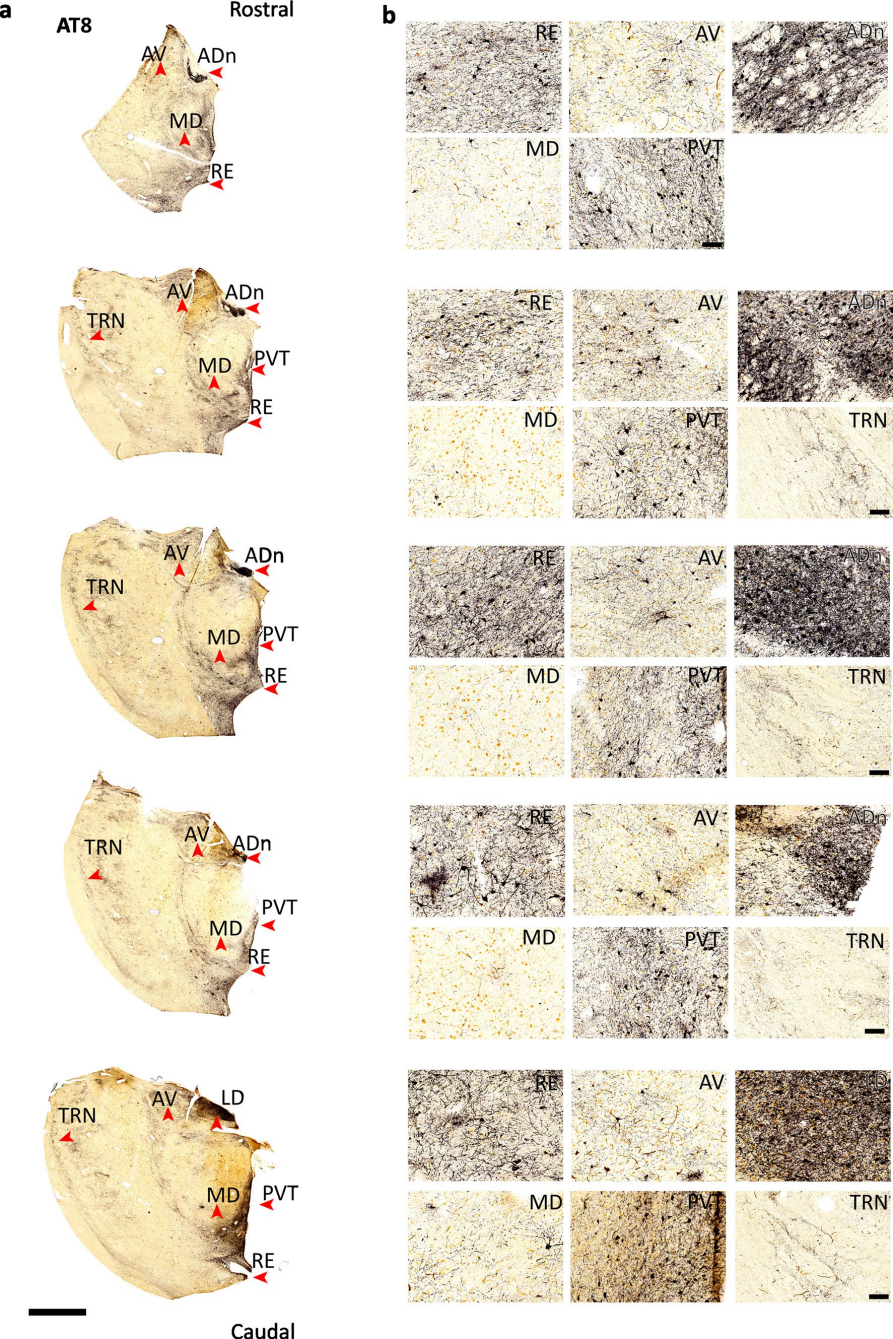
Serial sections of rostral thalamus for Braak stage VI. **a**,**b** Serial sections showing the distribution of ptau (AT8) at different rostral–caudal levels by DAB-HRP immunoreaction (nickel intensified, gray/black). Sections are 100 µm apart (Case 17, Braak stage VI). Scale bars: 4 mm (**a**), 100 µm (**b**)

**Fig. 4:**
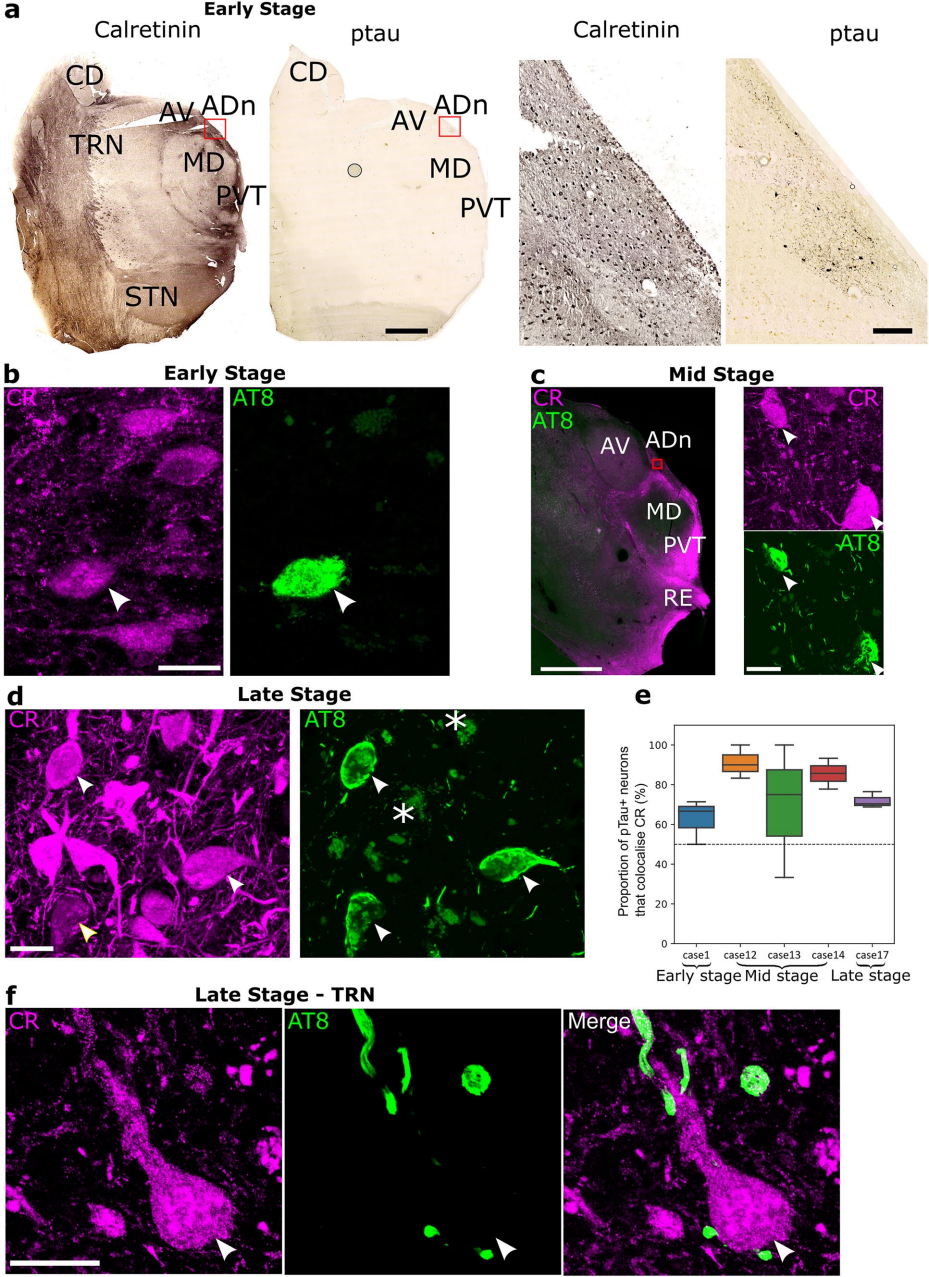
Ptau-containing neurons in the anterodorsal thalamic nucleus express calretinin. **a** Brightfield images of the rostral thalamus at Braak stage 0 (Case 1, FFPE, 10-µm-thick section). Left, adjacent sections immunoreacted for calretinin (CR) and AT8 (ptau) visualized with HRP-based DAB reaction end product. Right, enlarged view of the ADn (red boxed region) revealing CR+ and ptau+ neurons. **b**–**d** Subpopulations of calretinin (CR) immunoreactive neurons (magenta) colocalize ptau (AT8, green; white arrowheads) in the ADn. **b** Early stage (Case 1, FFPE sample), confocal z-projection (5.7 µm thick). **c** Middle stage (Case 12, perfusion-fixed, 50-µm-thick section). Left, widefield fluorescence. Right, detail CR and AT8 colocalization in the ADn from an adjacent section (red boxed region), confocal z-projection (3.7 µm thick). **d** Late stage (Case 17, perfusion-fixed, 50-µm-thick section), confocal z-projection (3.9 µm thick). Faded green signal is lipofuscin (e.g., asterisks). **e** Box plots of the percentage of AT8 immunoreactive cells in the ADn that were immunopositive for CR. **f** In the TRN, a CR+ neuron in close apposition to ptau+ boutons lack ptau (arrowhead, soma). Confocal z-projection, 2.2 µm thick (**c**). Scale bars: 4 mm (**a** left, **c** left); 250 µm (**a** right); 100 µm (**c** right); 20 µm (**b**, **d**, **f**). CD, caudate nucleus; STN, subthalamic nucleus

We examined post-mortem brain samples from 26 cases, grouped by the Braak tau stages of disease progression (Table 1) [16]. We assessed early (Braak stages 0–II; *n* = 15), middle (Braak stages III–IV, *n* = 6), and late (Braak stages V–VI, *n* = 5) cases, comparing the rostral thalamus (*n* = 25 cases) to the cortex (hippocampal formation and cingulate areas) and to other subcortical areas (Fig. 1a–f, 2, 3, 4; Tables 1, 2; Tables S3, S4).

We quantified ptau coverage and ptau-immunoreactive (ptau+) cells using the AT8 antibody, which recognizes phosphorylated serine 202 and threonine 205 residues of tau [34], in 50-µm-thick sections from perfusion-fixed human brains using a pixel classifier (Fig. 1g, h; Figure S1; Table 1; Methods). We confirmed these data with a ptau-intensity scoring system in a larger number of samples comprising the perfusion-fixed sections and 10-µm-thick FFPE sections and FFIF sections (Fig. 1i, j; Table S3; *n* = 26 cases).

We observed ptau+ neurons and processes restricted to the ADn in controls (‘pre-Braak’ stage 0; *n* = 4/6 cases; coverage = 0.56%, ptau+ cells = 1.29 mm^−2^, intensity score = 1; Figs. 1a, g–i, 4a; Table 2). In contrast to the ADn, when we examined all early-stage cases (Braak stages 0-II, *n* = 15 cases), we found that the other rostral thalamic nuclei, even those adjacent to the ADn, distinctly lacked ptau (Figs. 1a, 2a; Figure S2a; Table 2; Tables S3, S4). Among the postsynaptic targets of the ADn [65, 66], both the EC and retrosplenial cortex (RS; BA 30, 29 and 26), but not the presubiculum (PreS), exhibited sparse ptau (EC score = 1, *n* = 3 cases, RS score = 0.5, *n* = 2 cases; Fig. 1b, j; Table S3). Mild tau pathology in CA2 (*n* = 2/3 cases) suggests the presence of four-repeat tau isoforms or primary age-related tauopathy (PART) [40, 82] in parallel with ptau in the ADn.

At middle stages (Braak stages III-IV, *n* = 6 cases), the ADn contained moderate levels of ptau (coverage = 8.54%, ptau+ cells = 11.65 mm^−2^, intensity score = 2.65; Figs. 1c, g–i, 2; Figure S3a, b; Table 2; Tables S3, S4), whereas other rostral thalamic nuclei showed sparse ptau (AV: coverage = 0.15%, ptau+ cells = 14.49 mm^−2^, score = 0.5; paraventricular nucleus (PVT): coverage = 0.96%, ptau+ cells = 1.91 mm^−2^, score = 1.38; Fig. 1c, g–i; Tables S3, S4). In the cortex, ptau was observed at moderate-to-intense levels, with the RS, EC and prosubiculum (ProS) showing intense ptau immunoreactivity; the PreS displayed sparse immunolabeling at this stage (RS score = 3; EC score = 3; ProS score = 3; PreS score = 1; Fig. 1d, j; Table S3). In the RS, ptau was concentrated in the granular areas (BA 29 and 26).

At late stages (Braak stages V–VI), which included cases with Alzheimer’s disease (Table 1) [16], intense ptau was observed in the ADn, with a high density of ptau+ cells (*n* = 4/4 cases; coverage = 36.31%, ptau+ cells = 118.89 mm^−2^, intensity score = 3; Figs. 1e, g–i, 3; Tables S3, S4). The laterodorsal nucleus (LD) showed a high ptau density in the late stages, matching that of the ADn (Figs. 1i, 3; Table S3). The PVT had a similar ptau coverage to middle-stage ADn (*n* = 5/5 examined cases; 11.62%, score = 1.38; Figs. 1c, e, g, i, 3; Table 2; Tables S3, S4) and had the highest ptau+ cell count after the ADn (29.13 cells mm^−2^). The reuniens nuclear complex (RE) was similarly affected (score = 2). In contrast, the AV had lower coverage and ptau+ cell counts (coverage = 6.28%, ptau+ cells = 14.49 mm^−2^, intensity score = 1) followed by the mediodorsal nucleus (MD), which remained relatively sparse compared to the other examined nuclei (coverage = 1.62%, ptau+ cells = 2.84 mm^−2^, score = 1.25; Fig. 1e, g–i; Table 2; Tables S3, S4). The GABAergic thalamic reticular nucleus (TRN) lacked ptau immunopositive cell bodies; only axons and axon terminals were ptau+ (coverage = 2.98%, ptau+ cells = 0 mm^−2^, score = 2 Figs. 1e, g–i, 3; Tables S3, S4), consistent with previously published data [76]. In the cortex, ptau severely affected each examined area (Fig. 1f, j; Table S3), consistent with previous studies [16].

To provide additional context for the very early subcortical tau pathology in the ADn, we also examined the LC, which is susceptible to ptau at ‘pre-Braak’ stages [29, 33]. At early stages, in contrast to the sparse–moderate levels of ptau in the ADn, ptau expression in the LC was predominantly very weak and localized to processes resembling axons (*n* = 7/8 trace inclusions or sparse; *n* = 1/8 lacking ptau; *n* = Figure S4a, b; Table 2) [19]. In the middle and late stages, there was moderate ptau in somata, dendrites and axons (Figure S4c; Table 2). Interestingly, we detected neuromelanin in a few ptau+ somata. The DRn also showed moderate ptau in one tested Braak stage II case (Figure S4b). The lateral mammillary nucleus (LMB), which is presynaptic to the ADn (Fig. 1a) [80], lacked ptau in *n* = 3/4 early-stage cases (Figure S5a; Table 2). In one Braak stage II case, the ADn showed moderate-dense ptau and the LMB showed mild–moderate ptau (Case 23, Figure S5b). In middle and late stages, moderate–dense ptau was present in the LMB (Figure S5c, d; Table 2). The adjacent medial mammillary nucleus (MMB) and tuberomammillary nucleus had a similar pattern to the LMB (Figure S5b; Table 2). The data largely confirm previous reports [7, 16, 29, 33, 75].

In addition to neuronal ptau, we observed ptau+ ‘coiled bodies’ in the ADn (Figure S3c). Based on their size (~10 µm) and shape, we suggest that coiled bodies are localized to oligodendrocytes, which are typically overlooked in Alzheimer’s disease [55, 81]. We also detected ptau+ tufted astrocytes in six cases, which are associated with aging, Alzheimer disease, and other tauopathies [10, 46, 63]. Despite widespread ptau in astrocytes of varying shapes, sizes, and locations, including within the ADn (Figures S3a, b, d, S5c), neuronal ptau was consistently detected within the ADn, presynaptic LMB, and postsynaptic RS, suggesting this pathway can develop tau pathology in parallel with aging-related tau astrogliopathy (ARTAG) [46].

These results reveal that distinct nuclei of the rostral thalamus are affected early on by ptau, with the ADn consistently having the highest ptau density and ptau+ cells across all stages (Figs. 1a, 4a; Figures S2a, S3a; Table 2; Tables S3, S4).

### Calretinin-expressing neurons accumulate ptau in the rostral thalamus

We noticed that thalamic nuclei vulnerable to ptau were in CR-enriched regions (Figs. 2, 3, 4a) [31] and hypothesized that CR+ neurons were sensitive to accumulating ptau. We performed double immunolabeling with CR and AT8 (for ptau) and observed colocalization in neurons within the ADn, PVT, and RE (Fig. 4a–d). In the TRN, CR-enriched neurons lacked ptau (Fig. 4f), consistent with the distinct lack of ptau+ TRN cell bodies (Fig. 1h). As the ADn contained ptau+ neurons even in control cases (Figs. 1a, h, 4a), we tested whether CR+ neurons were affected at early, middle and/or late stages. Even at Braak stage 0, CR was detected in the majority of ptau+ neurons (64.3% CR+ , *n* = 1 case; Fig. 4a, b, e). In the middle stage, a large proportion of ptau+ cells were CR+ (81.1%; *n* = 3 cases; Fig. 4c, e), and in the late stage, 71.1% were CR+ (*n* = 1 case; Fig. 4d, e). In conclusion, CR-expressing neurons were affected very early on, and at every stage, the majority of ptau immunopositive cells were CR+ in the ADn.

### Subcellular distribution of ptau in the anterodorsal thalamus

**Fig. 5:**
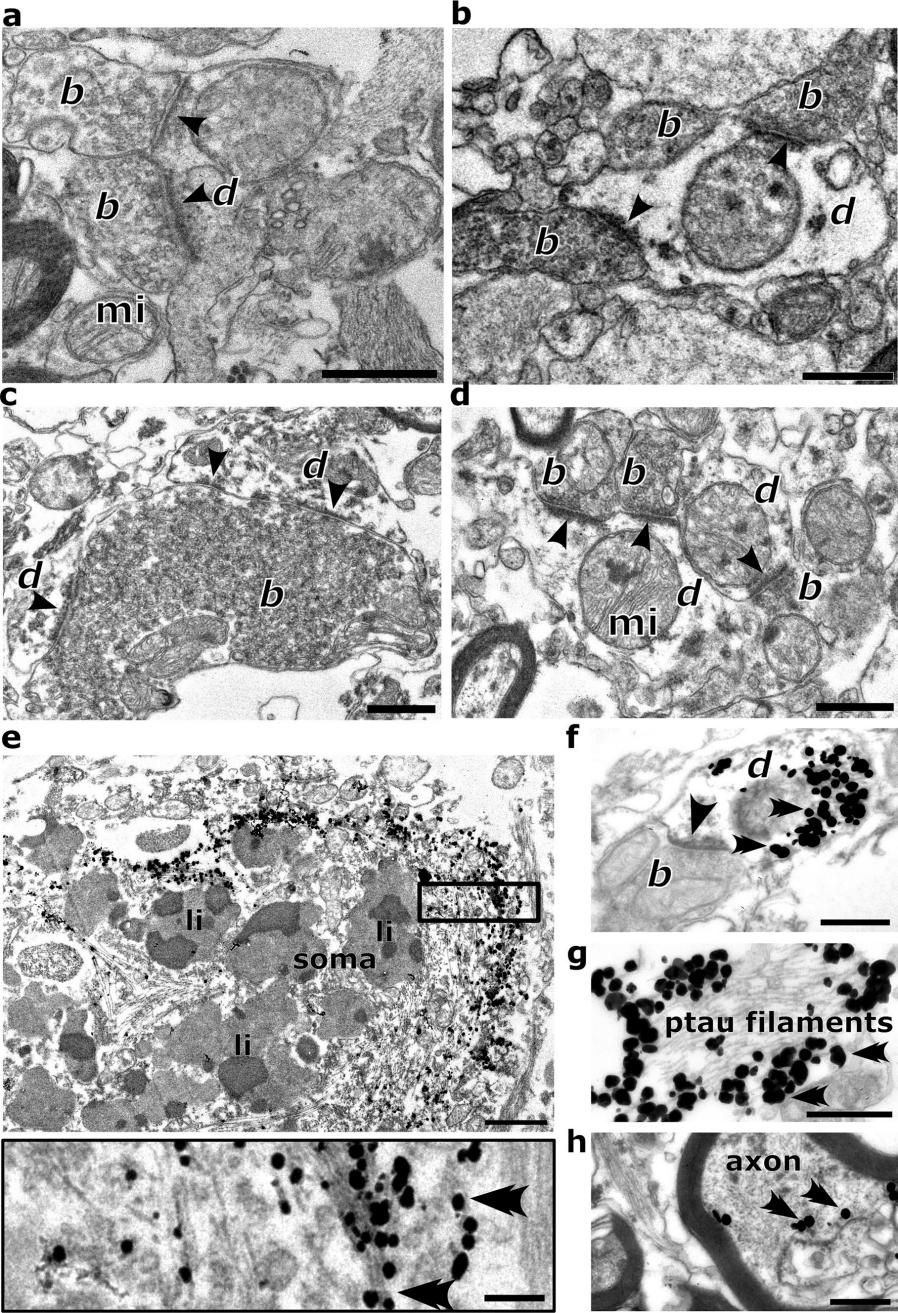
Electron micrographs of synaptic elements and tau filament-bearing structures in the human anterodorsal thalamic nucleus. **a**–**d** Unlabeled synaptic boutons (*b*) and dendrites (*d*) forming synaptic junctions (arrowheads): **a**,**b** early stage (Braak stage 0, Cases 3 and 4); **c** middle stage (Braak stage III, Case 12); a large bouton containing a high density of synaptic vesicles forms multiple synapses with its postsynaptic partners; **d** late stage (Braak stage VI, Case 17), small terminals form junctions (arrowheads) with small caliber dendrites. **e**–**h** Immunolabeling for ptau (AT8, gold–silver particles, all from Case 17, stage VI): **e** localization of ptau in a cell body containing lipofuscin (li); inset, detail of the boxed region showing immunolabeled ptau filaments (double arrowheads mark gold–silver particles); **f** a thin, ptau+ dendrite receiving an asymmetric synapse (arrowhead) from a small bouton (**b**); **g** high power image of paired helical filaments associated with silver intensified immunogold particles demonstrating ptau immunoreactivity; **h** ptau immunolabeling in a myelinated axon. Mitochondrion, mi. Scale bars: 0.5 µm (**a**–**h**), 0.25 µm (**e** inset)

After establishing that ADn neurons were especially vulnerable to ptau, we investigated the subcellular distribution of ptau to reveal how it spreads. To define synaptic structures at different stages of tau pathology, we examined ultrathin (~50–70 nm) sections of the ADn. We obtained electron microscopic samples from 4 cases (Cases 4, 12, 25, 17) that were appropriately preserved for quantitative analysis (Fig. 5a–d; Braak stages 0, II, III, and VI). We identified two main types of synaptic boutons with asymmetric synapses: large ~1–8 µm boutons (Fig. 5c; Figure S6a–c), consistent with presynaptic axon terminals from the mammillary body [68], and small <~1 µm diameter boutons (Fig. 5d; Figure S6a–c), resembling ‘classical’ cortical presynaptic terminals [64]. Some presynaptic boutons from stage 0 (*n* = 20/70), from stage II (*n* = 3/106) and stage VI (*n* = 31/103) had a highly electron opaque (‘dark’) appearance, ranging from a homogeneous state to others with recognizable vesicles and mitochondria, but all showing collapsed, scalloped forms (Figure S2c). This may indicate degeneration of certain nerve terminals, and/or be a sign of selective vulnerability to post-mortem/fixation conditions [24, 71]; these terminals were omitted from our quantification.

To identify subcellular ptau, we first examined cell bodies in the ADn, which contained abundant filaments (Fig. 5e). These resembled filaments previously found in the cortex of tauopathies including Alzheimer’s disease [9, 44, 74]. We visualized ptau with silver-enhanced immunogold particles, and observed that ptau was specifically associated with the intracellular filaments (Fig. 5e, g; Figure S2g), thus unequivocally demonstrating the association of ptau with the originally described paired helical filaments [45] at the ultrastructural level. Cell bodies also contained abundant lipofuscin (Fig. 4d, 5e). Filaments immunolabeled for ptau were also localized to dendrites (Fig. 5f; Figure S2d, g, h), and could be observed in large bundles (>1 µm) (Fig. 5g). Filament bundles were immunolabeled predominantly on the cytoplasmic surface, most likely due to reagents not penetrating into the bundle (Fig. 5g). We also detected ptau in myelinated axons (Fig. 5h). Given that ptau was localized to a variety of subcellular domains, we next investigated whether ptau can also be associated with axon terminals in the ADn.

### Subcortical vesicular transporter 2-expressing presynaptic terminals preferentially contain ptau

**Fig. 6:**
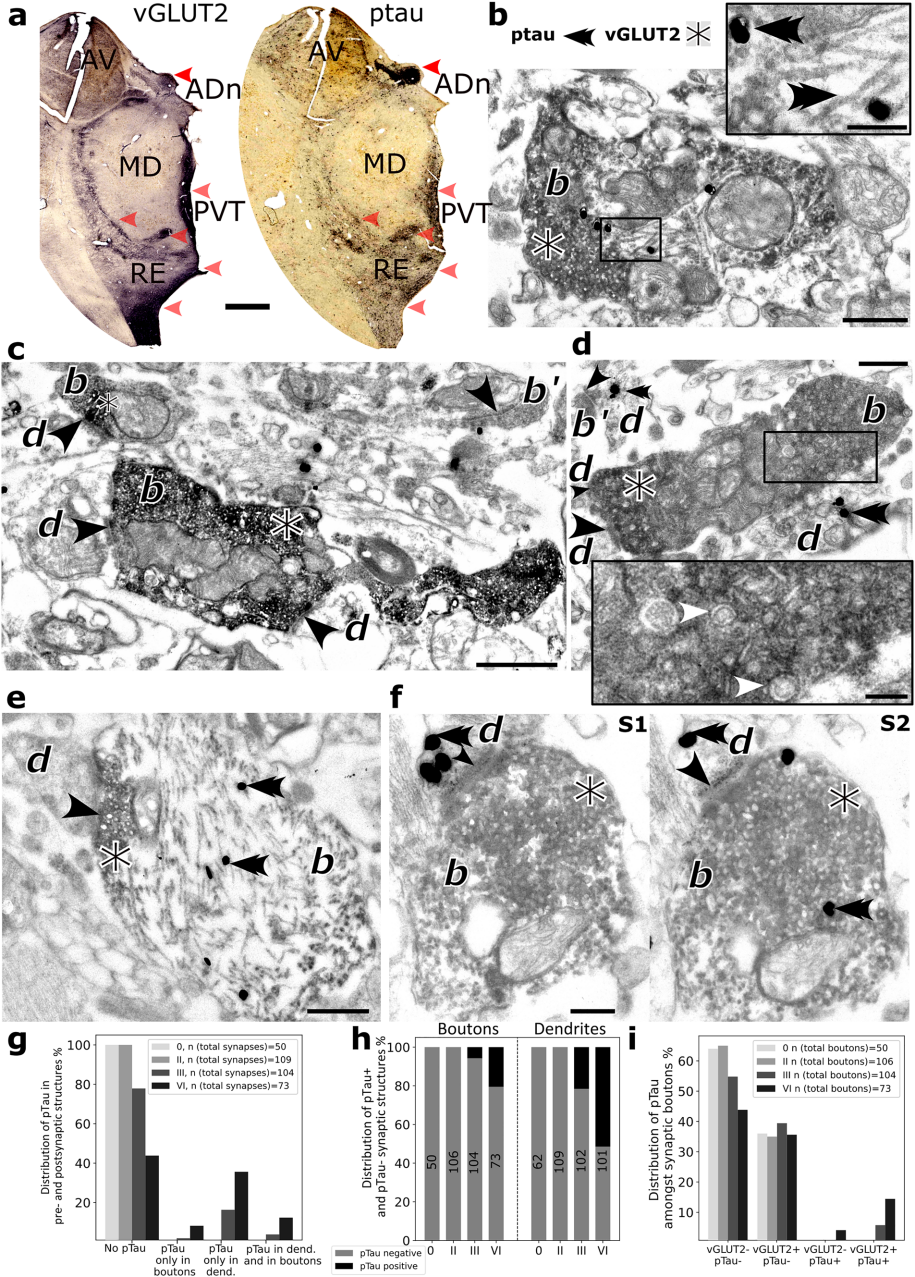
Localization of ptau to vGLUT2-expressing presynaptic terminals. **a** Brightfield microscope images of rostral thalamus (serial sections, late stage, Case 17) reacted for vGLUT2 (left) and ptau (AT8, right); HRP-based diaminobenzidine end products (DAB). Note similar distributions for vGLUT2 and ptau (red arrowheads). **b**–**f** Electron micrographs showing ptau filaments (AT8 immunolabeling, e.g., double arrowheads mark gold–silver particles) and vGLUT2-immunolabeled boutons (DAB) from the ADn of Case 17. Abbreviations: *b*, bouton; *d*, dendrite. Black arrowheads mark synaptic junctions. **b** A vGLUT2-immunopositive (vGLUT2+) bouton containing ptau filaments in the middle. Inset, higher magnification of helical ptau filaments from boxed region. **c** Two vGLUT2+ boutons (DAB) and a vGLUT2- bouton (*b*′, top right) lacking ptau, a silver particle is adjacent to the bouton. **d** A large vGLUT2+ terminal **b** containing double-walled vesicles and clumped mitochondria. Inset, detail of boxed region showing vesicles (e.g., marked by white arrowheads). A small bouton (*b*′, top left) forms a synapse with a ptau immunopositive dendrite. **e** A large vGLUT2+ bouton (note DAB end product on vesicles at the synapse) containing dense ptau filaments. **f** Electron micrographs of sequential sections (S1, S2) showing ptau in both the vGLUT2+ bouton and the postsynaptic dendrite. Double arrowheads mark examples of gold–silver particles; asterisks label dark DAB product around vGLUT2+ vesicles. **g** Proportion of synapses (%) in the ADn with or without ptau in the presynaptic bouton and/or postsynaptic dendrite at Braak stages 0, II, III, VI. **h** Proportions of synaptic boutons (%, left) and dendrites (right) containing ptau at the different stages. **i** Proportion of synaptic boutons (%) with or without vGLUT2 and ptau immunoreactivities at the different stages. Scale bars: 2 mm (**a**), 0.5 µm (**b**, **d**, **e**), 1 µm (**c**), 0.25 µm (**f**), 150 nm (**b**,**d** insets)

Large presynaptic terminals of subcortical origin contain vesicular glutamate transporter 2 (vGLUT2) [59]. We observed strongly overlapping distributions of vGLUT2 and AT8 immunoreactivities at the light microscopic level, especially in the ADn, RE, PVT, and internal medullary lamina (Fig. 6a). The overlapping vGLUT2 and AT8 distributions suggested that vGLUT2 may be related to ptau. When we examined sections immunoreacted for both vGLUT2 and AT8, we discovered that ptau was localized to vGLUT2+ boutons (Fig. 6b, e, f; Figure S2b, d). Whereas some vGLUT2+ boutons showed no signs of abnormalities (Fig. 6c), others were degenerating (Fig. 6d; Figure S2c). The degenerating vGLUT2+ boutons had clumped mitochondria (Fig. 6d). Many of these boutons contained large (80–100 nm) double-walled vesicles (Fig. 6d) [44], consistent with autophagy or the packaging and/or potential release of different forms of tau [26, 56]. Not all vGLUT2-positive degenerating boutons displayed detectable ptau, at least in the sections that we examined. In some degenerating boutons which were immunoreactive for both vGLUT2 and ptau, ptau+ bundles of filaments occupied a large proportion of the volume crowding out vesicles (Fig. 6b, e), which may cause impairments in neurotransmission. We also observed synaptic partners consisting of presynaptic vGLUT2+ boutons and postsynaptic dendrites that *both* contained ptau (Fig. 6f; Figure S2d), suggestive of transsynaptic spread between the mammillary bodies and ADn (Fig. 1a; Figure S5b). The size distribution of ptau-positive terminals also confirmed that large presynaptic terminals are preferentially affected by ptau pathology (Figure S6a–c).

### The distribution of presynaptic and postsynaptic ptau suggests transsynaptic spread

Given the observation of ptau in both presynaptic terminals and postsynaptic dendrites (Fig. 5f, 6b, d–f; Figure S2b, d, g, h), we quantitatively characterized how the synaptic distribution of ptau changed across different Braak stages, examining 652 presynaptic boutons and postsynaptic dendrites, each of which was followed over several serial sections.

At Braak stage 0, despite ptau being detectable at the light microscopic level (Figs. 1a, 4a), all sampled boutons and dendrites lacked ptau (*n* = 50/50 boutons, *n* = 62/62 dendrites; Case 4; Fig. 6g, h). Similarly, despite abundant ptau in the ADn at Braak stage II (Fig. 1i; Figure S2a; Table 2; Table S3), we did not detect ptau in boutons (*n* = 106/106) or dendrites (*n* = 109/109) at the electron microscopic level (Fig. 6g, h; Figure S2e, f), probably due to the limited sampling area.

At Braak stage III, 5.8% of boutons (*n* = 6/104) and 21.6% of dendrites (*n* = 22/102) were ptau+ (Case 12; Fig. 6g, h; Figure S2d). In this stage, the proportion of synapses in which *both* the presynaptic boutons and the associated postsynaptic dendrites contained ptau was 3.9% (*n* = 4/104; Figure VGLUT2f S2d). At Braak stage IV, we detected ptau within dendritic appendages in close apposition to vGLUT2+ terminals, which also contained ptau (Figure S2b).

At Braak stage VI, the proportion of affected boutons and dendrites greatly increased: 20.6% of boutons (*n* = 15/73) and 51.5% of dendrites (*n* = 52/101) contained ptau (Case 17; Fig. 6b, d–h). Furthermore, 12.3% (*n* = 9/73) of synapses consisted of *both* ptau+ boutons and ptau+ dendrites (Fig. 6f, g; Figure S2d). These data demonstrate that the proportions of both the presynaptic and postsynaptic elements containing ptau increase with Braak stage.

Finally, we examined the relationship between presynaptic vGLUT2 and ptau across stages. At the early stage, we identified vGLUT2+ boutons (*n* = 18, Braak stage 0; *n* = 37, Braak stage II; Figures S2e, f, S6d), but did not detect ptau (Figure S6). But by the middle stage, from a total of 104 synaptic boutons, 5.8% (*n* = 6) were both vGLUT2 and ptau double immunopositive (Fig. 6i), whereas none of the vGLUT2 immunonegative boutons (*n* = 57) were ptau+. In other words, 100% of ptau+ boutons were vGLUT2+ (*n* = 6) and 12.8% of vGLUT2+ boutons (*n* = 47) were ptau+, supporting the hypothesis of selective vulnerability of subcortical vGLUT2+ synaptic terminals. Filamentous contacts with postsynaptic structures, known as puncta adherentia, are associated with mammillothalamic terminals [59]. We identified puncta adherentia between vGLUT2+ boutons and postsynaptic dendrites containing ptau (Figure S2d). Moreover, the small corticothalamic boutons lacked ptau (Figure S6b, e), which indicates that ptau in the ADn is unlikely to have spread anterogradely from the cortex.

**Fig. 7:**
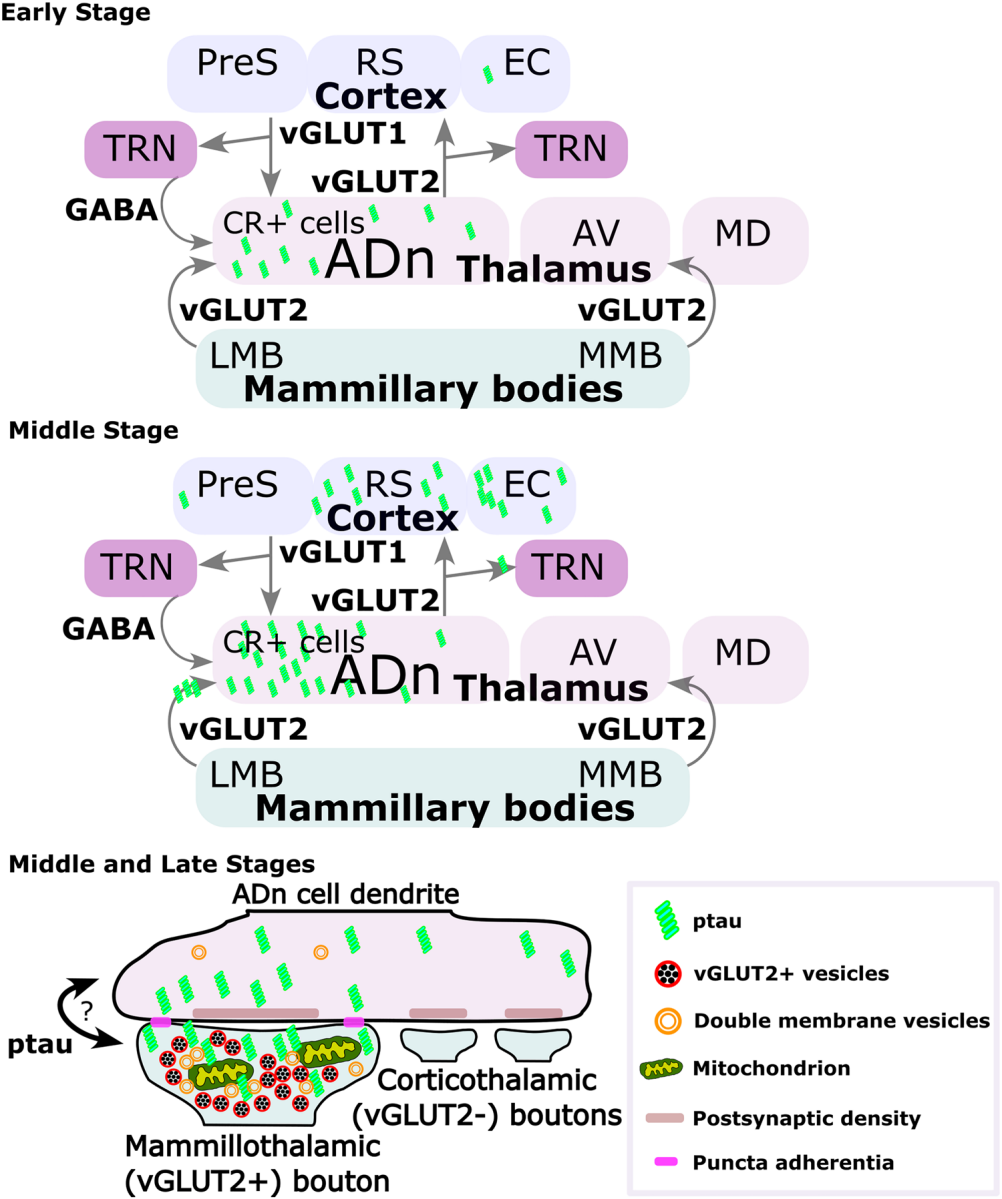
Schematic of ptau transsynaptic spread involving vGLUT2+ terminals in the ADn. Scheme summarizing the data. According to our hypothesis, in the early stage (top), ptau (green) initially accumulates in CR+ cells of the ADn. By the middle stage, ptau has accumulated in cortical areas postsynaptic to the ADn, mainly the granular RS and EC. In the thalamus, ptau is present in ADn axons innervating the TRN and in mammillothalamic (vGLUT2+) terminals innervating the ADn. The GABAergic cells in the TRN are not invaded by ptau, nor are the corticothalamic terminals in the ADn. Bottom, details of ptau within the ADn at the middle stage (also applies to the late stage). Transsynaptic spread between the large vGLUT2+ mammillothalamic boutons and ADn dendrites may be retrograde or anterograde, potentially mediated through puncta adherentia. Puncta adherentia are not found between small corticothalamic boutons and postsynaptic dendrites, and ptau was not observed in these presynaptic terminals at either middle or late stages, despite ptau in presynaptic cortical areas. See “Discussion” for further details

In the late stage, out of a total of 73 synaptic terminals, an even higher proportion showed vGLUT2 and ptau colocalization (16.4%; *n* = 12; Fig. 6i), i.e., ~*80%* of ptau+ boutons (*n* = 15) were immunopositive for vGLUT2. And of all vGLUT2+ boutons (*n* = 38), 31.5% were ptau+. The data on the colocalization of vGLUT2 and ptau is even likely to be an *underestimate*, given that large ‘dark’ boutons are likely to be degenerating mammillothalamic terminals (Figures S2c, S6f), and we only sampled relatively few sections for each terminal. The above results suggest that vGLUT2+ boutons are strong candidates for the transsynaptic spread of ptau between postsynaptic ADn neurons and presynaptic mammillary body neurons within the Papez circuit (Fig. 7).

## Discussion

### Significance of ADn tau pathology

Untangling which brain regions are first affected in neurodegenerative diseases may facilitate earlier diagnosis and enhance treatment options. We found that the ADn accumulated ptau very early on at the pre-cortical stage that lacked significant neocortical or hippocampal pathology (Braak stage 0). Calretinin-expressing neurons of the ADn were vulnerable at all disease stages. Given the consistently high ptau density and ptau+ cell counts that we observed in the ADn at all stages and in a variety of cases with different clinical and pathological hallmarks, we suggest that the human ADn is a ‘hub’ for early-forming tau pathology and may be part of PART [82], later degenerating in Alzheimer’s disease [85]. It would be informative to test whether the ADn contains ptau in younger individuals [17] and establish which forms of tau (e.g., soluble ‘pretangles’) are present [19]. Using perfusion-fixed tissue and immunogold labeling, we localized, to our knowledge for the first time, ptau to presynaptic boutons and postsynaptic dendrites, with paired helical filaments resembling those described previously in cases of Alzheimer’s disease and other tauopathies [9, 10, 44, 45, 49]. Surprisingly, despite the classical hypothesis of ptau spreading via corticocortical pathways originating in the EC [18], ptau was not found in corticothalamic boutons in the ADn. Instead, ptau was preferentially localized to large vGLUT2-expressing subcortical terminals. This supports the transsynaptic theories of disease progression but highlights an alternative or parallel major subcortical glutamatergic pathway necessary for driving the spread of ptau in relation to the emergence of Alzheimer’s disease. In contrast to the well-defined connectivity of the Papez circuit, the diffuse widespread projections of the LC make it difficult to explain why the very early accumulation of misfolded forms of tau would propagate preferentially from the LC to the EC and not to all other areas the LC projects to [18]. In addition, the vast majority of LC terminals do not form classical synapses in the cortex [13], and thus this terminal type would require a specialized mechanism for “transsynaptic” spread other than via classical synapses.

Lesions of the anterior nuclear group (ADn, AV, AM) impair spatial reference and working memory in rats [32, 50, 79, 83], and thalamic infarctions (especially those that include the mammillothalamic tract) are associated with amnesia in humans [78]. Also, in mice, selective disruption of the ADn impairs spatial working memory [60]. The anterior nuclear group shares cortical targets through direct projections to the granular RS, PreS, and EC [65, 66]. The AV and AM receive inputs from multiple cortical areas including the EC, yet the ADn only receives input from the dorsal part of the RS and not the EC [42, 62]. If ptau had spread directly from the EC to the anterior nuclear group, we would have detected early ptau in the AV, but it was sparse even at the middle stage. Another key difference between the anterior thalamic nuclei is that the ADn receives glutamatergic input from the LMB, whereas the AV and AM receive input from the MMB [35, 37].

The large vGLUT2-containing axon terminals that we found to selectively accumulate ptau likely arise from the LMB. They are thought to act as ‘drivers’, releasing glutamate in response to dynamic changes in the pattern of sensorimotor inputs evoked by stimuli, such as changes in head direction or shifts in gravity [47, 58, 59, 73]. Postsynaptically, ADn neurons will be strongly depolarized leading to high-frequency firing within the receptive field [73]. It is possible that the gradual and selective accumulation of ptau within vGLUT2+ axon terminals and neurons in the ADn (independent of the AV and AM) will disrupt receptive fields (e.g., head direction tuning, angular velocity) and firing rates, thereby decreasing the information content provided to postsynaptic neurons in the RS, PreS, and EC [65, 66]. This might cause early and progressive deficits in the awareness of orientation and a resulting increase in the probability of losing balance. Our findings may explain the early impairments in spatial navigation and orientation, path integration deficits, and an increased number of falls in people that go on to develop Alzheimer’s disease [5, 12, 22, 25, 38, 70].

### Propagation of ptau involving large subcortical vGLUT2-containing terminals

Nuclei adjacent to the ADn such as the AV and MD were relatively resistant to ptau, even in late stages, suggesting that in the thalamus, propagation of ptau is facilitated via specific synapses and circuits rather than geometrical proximity. Our data are consistent with the following hypothesis (Figure S-SCHEMATIC): ptau first accumulates in CR-expressing ADn neurons of the Papez circuit. Next, ptau spreads to the large mammillothalamic vGLUT2+ terminals from ADn neuron dendrites. Myelinated axons of the ADn neurons transfer ptau to vGLUT2+ terminals in the TRN and to cortical target areas such as the granular RS. A lack of ptau+ cell bodies in the TRN [76] at any stage suggests that the predominantly GABAergic cell population is ‘resistant’ to the spread of ptau from vGLUT2+ boutons, whereas cortical neurons postsynaptic to ADn neurons are likely to be vulnerable. Note that we cannot currently rule out ptau spreading retrogradely from cortical neuron dendrites into ADn vGLUT2+ terminals, then from ADn dendrites to mammillothalamic terminals at the early stages (Fig. 7). However, the very early appearance of ptau+ neurons in the ADn prior to cortical neurons makes this route unlikely. Anterograde spread of ptau from the cortex to the ADn is also not supported by our data due to the lack of ptau in small corticothalamic terminals at the middle stage or even in the late stage. Moreover, we did not detect ptau in the LMB at Braak stage 0 (when ptau was present in the ADn), ruling out anterograde spread from the LMB, at least in the cases we examined. Nevertheless, the vGLUT2+ mammillothalamic terminals are especially vulnerable due to a nearly threefold increase of ptau in these boutons from the middle stage to late stage. These boutons are unusual due to the accumulation of double-walled vesicles that may represent a type of autophagic/secretory organelle, and the presence of puncta adherentia, which contain intercellular adhesion proteins such as nectins. The puncta adherentia may even be required for the transfer of ptau (Fig. 7), and are located at other sites of potential ptau propagation including between mossy fibers of dentate granule cells and CA3 pyramidal neurons [6, 51]. In contrast, puncta adherentia are lacking in the corticothalamic terminals in the ADn, which did not contain ptau.

Data supporting the transsynaptic spread of tau has been obtained in animal models [4, 20, 27, 39, 48] and humans [23, 28], with presynaptic glutamate release inducing a rise in extracellular tau [86]. However, transsynaptic spread has not been previously demonstrated in the human brain at the subcellular level in well-preserved tissue. Tau may be released extracellularly (e.g., in vesicles) under certain conditions [67, 84], which may contribute to the prion-like spread [18, 21, 23]. The large vGLUT2+ terminals packed with double-walled vesicles resemble those found at a lower density in the cortex of Alzheimer’s disease cases [44, 49]. Due to their prime position within the terminal, we suggest these vesicles are candidates for the transport and/or release of tau. Alternatively, or in addition, ptau may be directly associated with vGLUT2-containing vesicles, consistent with a previous report of tau being associated with the cytosolic surface of vGLUT2+ synaptic vesicles [77].

### Selective vulnerability of different cell types

Identification of affected cell types in different neurodegenerative diseases is crucial for the understanding of biochemical factors that cause susceptibility and for therapy development, as it the case for dopaminergic neuronal loss in Parkinson’s disease [43]. Specific cell types may be more or less vulnerable due to their connectivity (e.g., presynaptic inputs, extensive axonal arbors), metabolic demands (e.g., maintaining high firing rates), or a combination. Noradrenergic LC neurons are thought to be vulnerable due to their dense cytoarchitecture and long-range axonal projections [33], which could also apply to the ADn. Wolframin-expressing neurons are susceptible to ptau in human EC, and in mice ptau may propagate from wolframin+ EC cells to CA1, which is linked to memory impairments [27]. We found that the calcium-binding protein CR was associated with ptau in the thalamus as early as Braak stage 0. This may represent a vulnerable subpopulation having specific connectivity with the cortex (e.g., with the RS or EC) and mammillothalamic tract, forming a key part of the Papez circuit, with specific conditions for propagation (e.g., via puncta adherentia). Future immunohistochemical and RNA sequencing studies may shed light on the specific molecular identity of this subpopulation. Finally, it is recognized that glial cells also accumulate different forms of ptau [10, 46, 55, 87], occurring in parallel with neuronal ptau in the cases we examined. Future studies will establish the contribution of different glial cell types to tau propagation, neurodegeneration, and their association with a disrupted neurogliovascular unit [54], which may be one of the underlying triggers for tau pathology in highly vulnerable brain regions such as the ADn. In summary, our results demonstrate that the connectivity, synaptic specificity, and molecular profile of subpopulations of glutamatergic rostral thalamic neurons relating to spatial orientation potentially drive progression of tau pathology in the human brain.

### Supplementary Information

Below is the link to the electronic supplementary material.

Supplementary file1 (PDF 16234 KB)
