# Supplementary material for: Early and selective localization of tau filaments to glutamatergic subcellular domains within the human anterodorsal thalamus
Source: Acta Neuropathol. 2024 Jun 11;147(1):98. doi: 10.1007/s00401-024-02749-3 (PMC11166832; doi:10.1007/s00401-024-02749-3)
Supplement: Supplementary file 1 — Supplementary file1 (PDF 16234 KB) [file 401_2024_2749_MOESM1_ESM.pdf]

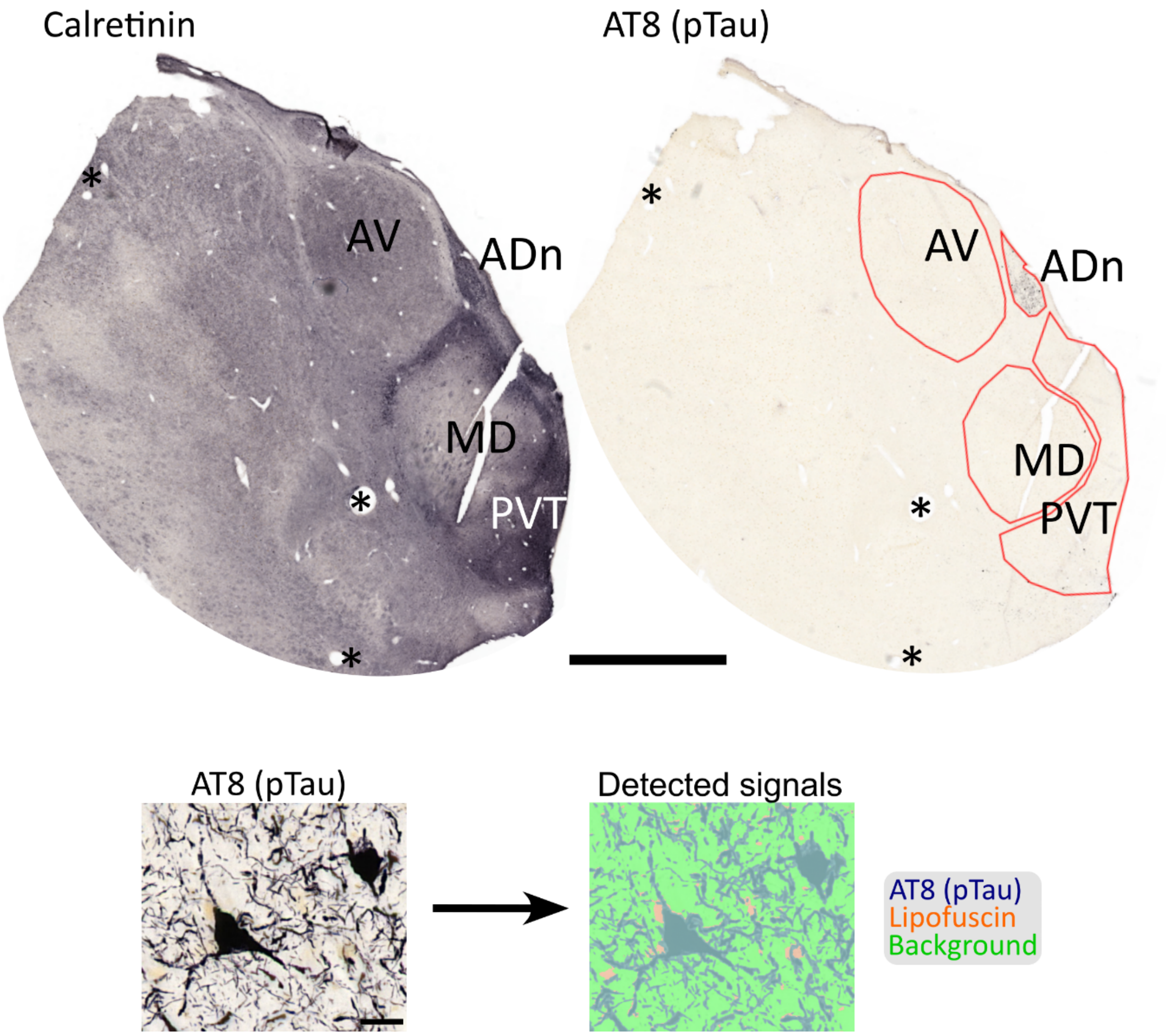

Figure S1. Pixel classifier.

Thalamic nuclei were delineated by CR immunoreactivity (left), followed by AT8 signal detection (right) using a pixel classifier (see Materials and Methods). Scale bars: 4 mm, 20 µm (enlarged ADn region).

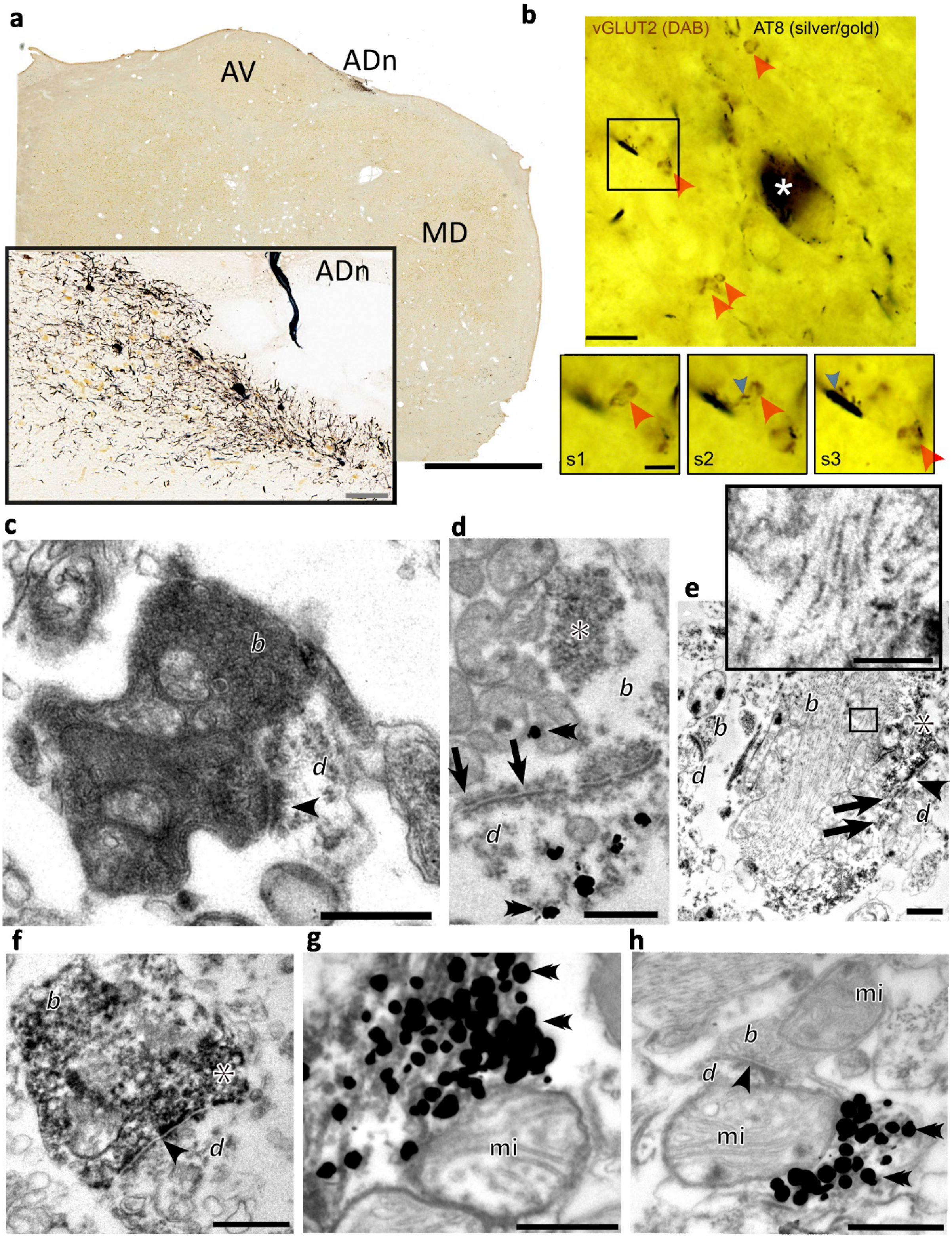

Figure S2. Light and electron microscopic localization of ptau to vGLUT2+ presynaptic terminals and postsynaptic dendrites in the ADn.

(**a**) Brightfield image of ptau (AT8 immunoreactivity; HRP-based DAB end-product) in a 50-µm-thick section of the rostral thalamus, showing ptau restricted to the ADn. Inset, ptau is localized primarily to neuronal processes in the ADn. Braak Stage II, Case 23. (**b**) Identification of ptau (AT8, black, gold-

silver particles) and vGLUT2+ terminals (HRP-based DAB end-product, brown, e.g. red arrows). Asterisk, a cell body containing ptau. Insets, consecutive optical sections (s1-s3) showing two vGLUT2+ boutons associated with ptau; 1 bouton innervates a ptau+ dendritic appendage (blue arrowhead). Braak stage IV, Case 14. (**c**) An electron opaque ('dark') bouton making a synapse (arrowhead) with a dendrite. Braak stage VI (Case 17). (**d**) Puncta adherentia (e.g. arrows) between a bouton and a dendrite. Asterisk, vGLUT2 (HRP-based DAB). Gold-silver particles (double arrowheads) recognizing ptau (AT8) were detected in both the vGLUT2+ bouton and in the dendrite. Braak stage III (Case 12). (**e**) A vGLUT2-immunopositive bouton (asterisks: dark DAB end-product around vGLUT2+ vesicles) containing filaments forming a synapse with a dendrite. Inset, detail of filaments. Braak stage II (Case 25). (**f**) A vGLUT2+ bouton forms a synapse with a dendrite. Braak stage II (Case 25). (**g-h**) Dendrites containing bundles of ptau+ filaments. Braak stage VI (Case 17). Dendrite, *d*; bouton, *b*; mitochondrion, mi. Scale bars: (a) 4 mm, 80 μm (inset); (b) 10 μm, 4 μm (insets); (c-h) 0.5 μm, 0.2 μm (e inset).

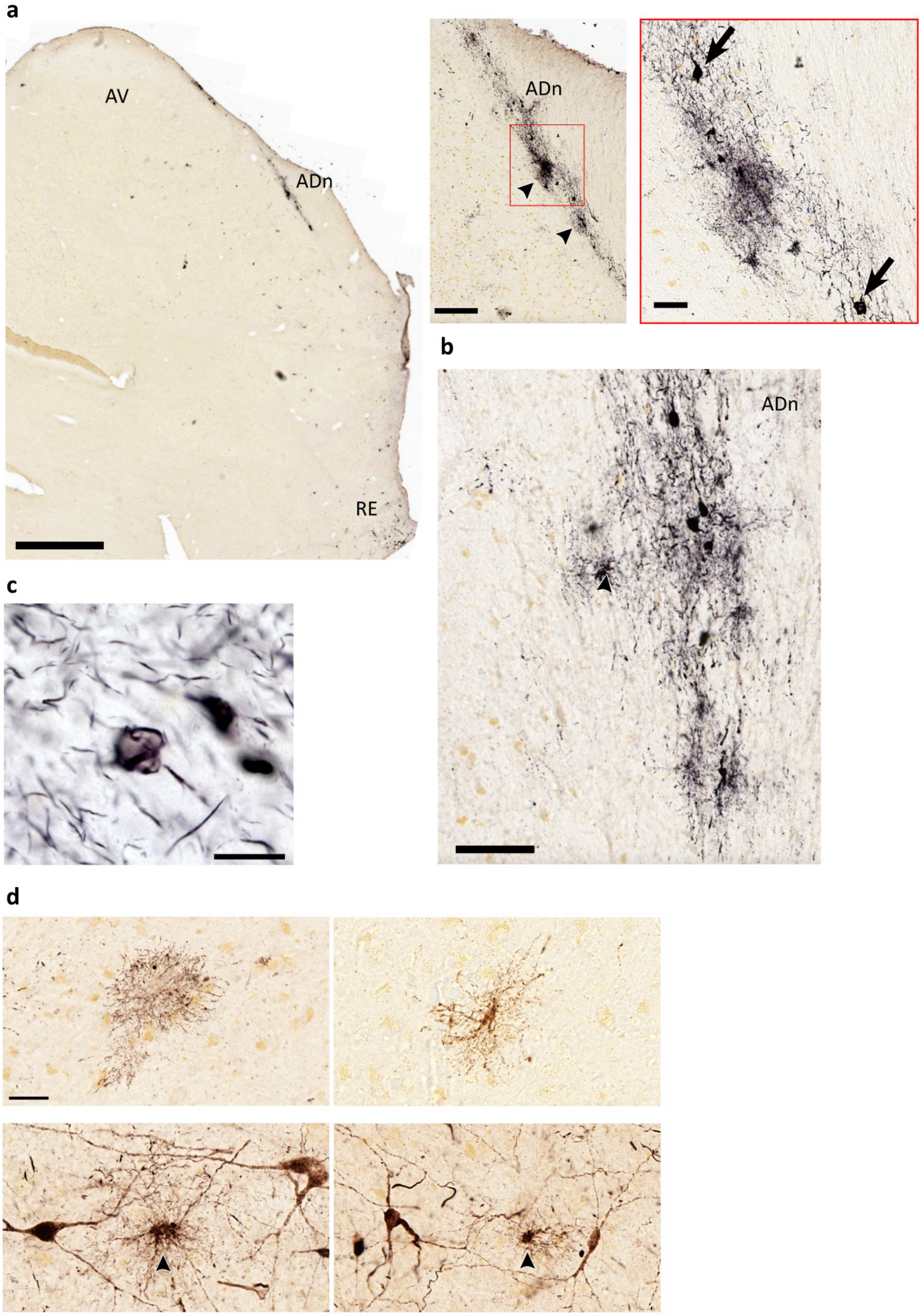

Figure S3. Glial cells containing ptau.

Brightfield images of neurons and glial cells, detected by HRP-DAB immunoreaction for AT8. (**a**) Tau pathology restricted to the ADn of a Braak stage IV case (Case 14). Right, detail of ADn. Enlarged

region shows neurons (e.g. arrows). Arrowheads, astrocytes. (**b**) Tau pathology in neurons and astrocytes (e.g. arrowhead) within the ADn in an adjacent section to that of (a). (**c**) A 'coiled body' in the ADn. Braak stage VI, Case 17. (**d**) A subpopulation of astrocytes were immunoreactive for ptau in the AV (top panels) and amongst immunopositive neurons in the posterior hypothalamic area (bottom panels; arrowheads). Braak stage III, Case 13. Scale bars: (a) 2 mm; 250 µm, 50 µm (a insets); (b) 100 µm; (c) 10 µm (c); (d) 40 µm.

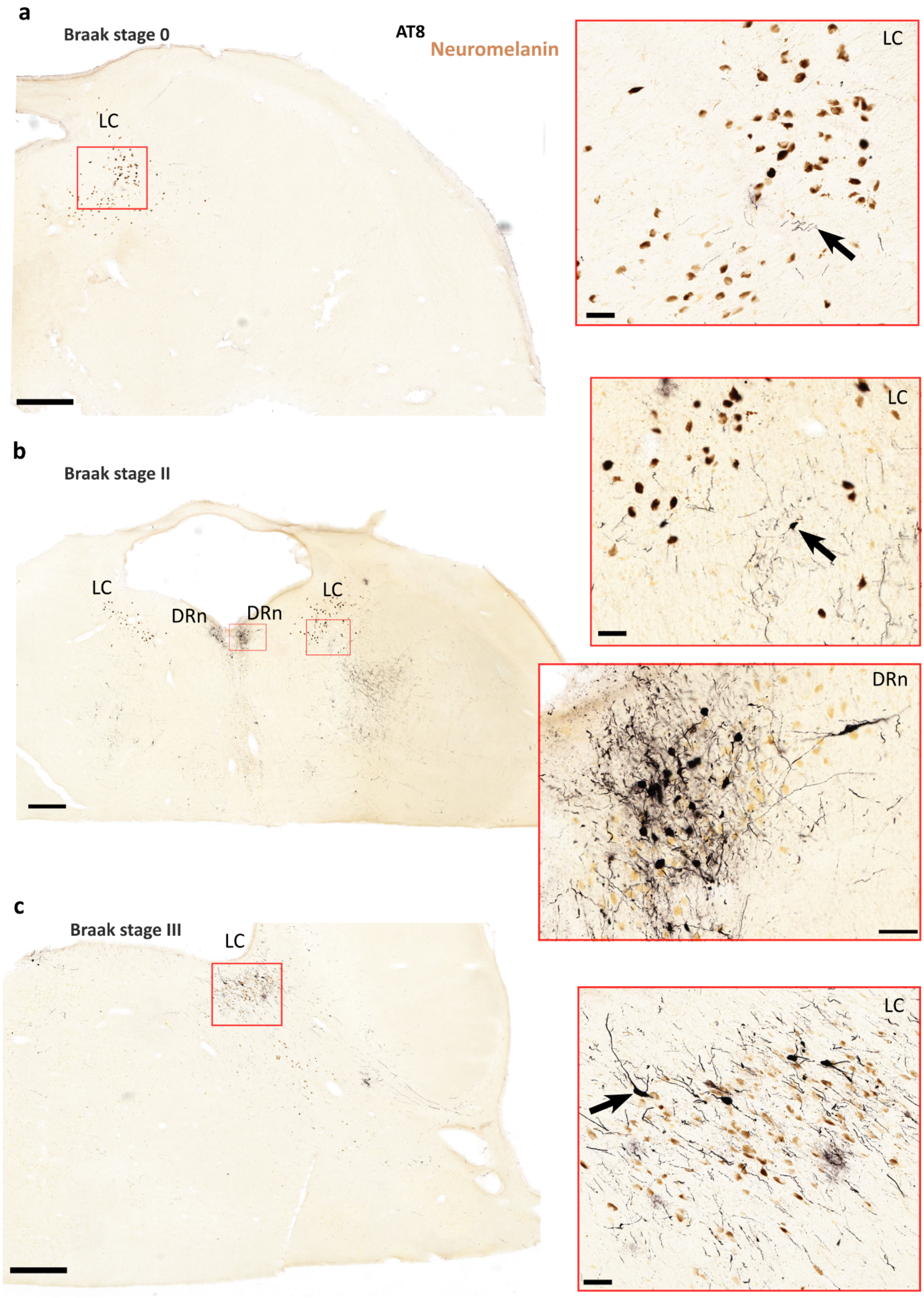

Figure S4. Tau pathology in the locus coeruleus and dorsal raphe nucleus in early-stage cases.

(**a-c**) Brightfield images of ptau (AT8 immunoreactivity; HRP-based DAB end-product) in 50-µm-thick sections of the midbrain. (**a**) Sparse ptau restricted to axons or dendrites (e.g. arrow) in the locus coeruleus at Braak stage 0 (Case 22). Note lack of ptau within cell bodies that contain neuromelanin (brown). (**b**) Sparse ptau at Braak stage II (Case 26). Few small ptau+ cell bodies are detectable (e.g. arrow). In contrast, the DRn contains a higher density of ptau+ neurons. (**c**) Sparse ptau at Braak stage III (Case 12). Some cell bodies contain ptau (e.g. arrow). Scale bars: 1 mm (main); 100 µm (insets).

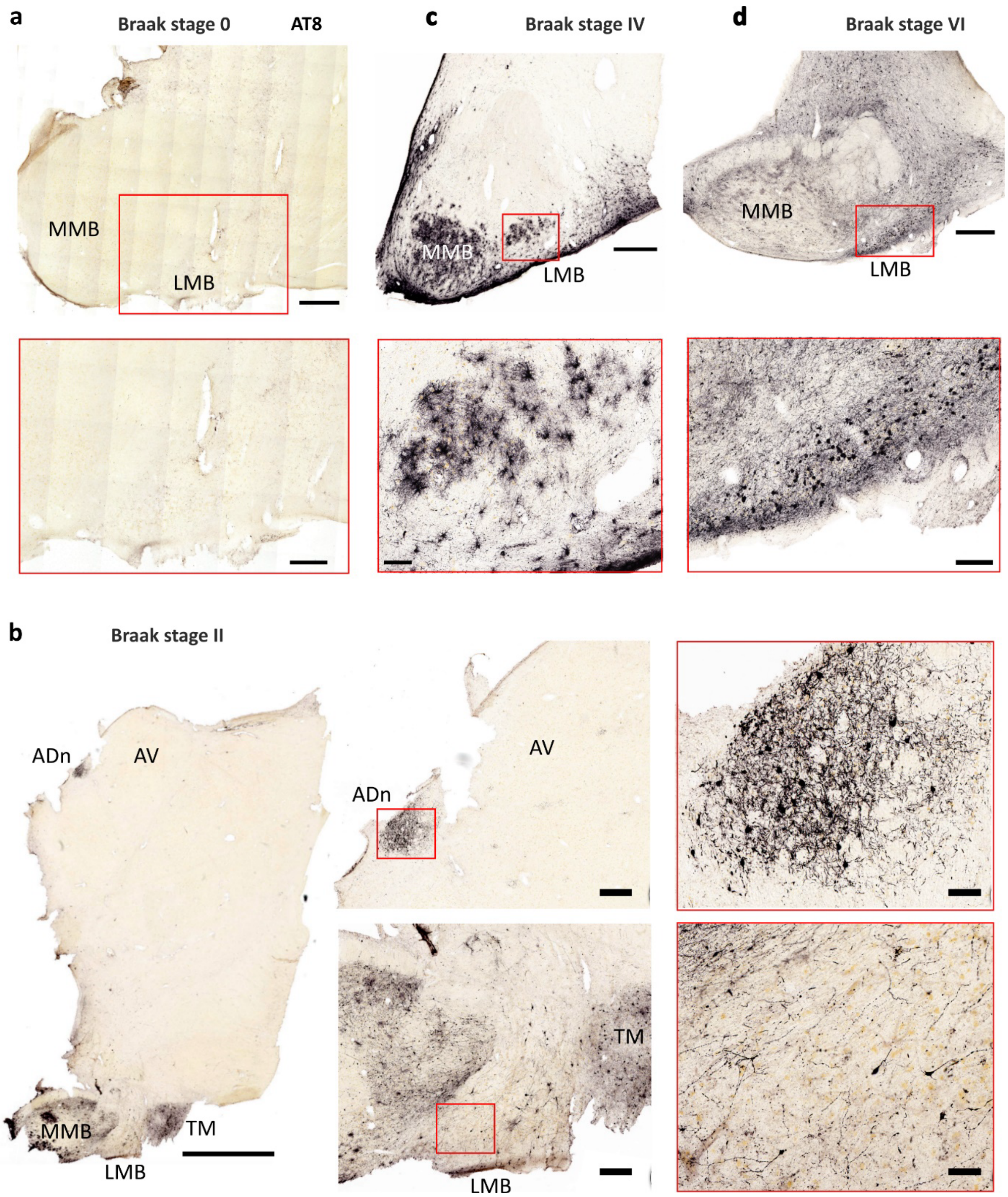

Figure S5. Accumulation of ptau in the mammillary body.

(**a-d**) Brightfield images of ptau (AT8 immunoreactivity; HRP-based DAB end-product) in 50-µm-thick sections containing the mammillary body. (**a**) Lack of detectable ptau in the mammillary body at Braak stage 0 (Case 24). Inset, detail of region centered on the LMB. (**b**) A section containing both the rostral thalamus and mammillary body, Braak stage II (Case 23). The ADn contains a high density of ptau whereas the presynaptic LMB contains mild-moderate ptau. Right, detail of ADn and LMB. The MMB and TM contain moderate-dense ptau. (**c**) Tufted astrocytes containing ptau within the MMB and LMB in a Braak stage IV case (Case 14). Inset, enlarged view of LMB. (**d**) Dense ptau covering the entire mammillary body at Braak stage VI (Case 17). Inset, enlarged view of LMB. Scale bars: (a) 800 µm, 400 µm (inset); (b) 5 mm, 500 µm (middle insets) and 100 µm (right insets); (c) 800 µm, 100 µm (inset); (d) 1 mm, 250 µm (inset).

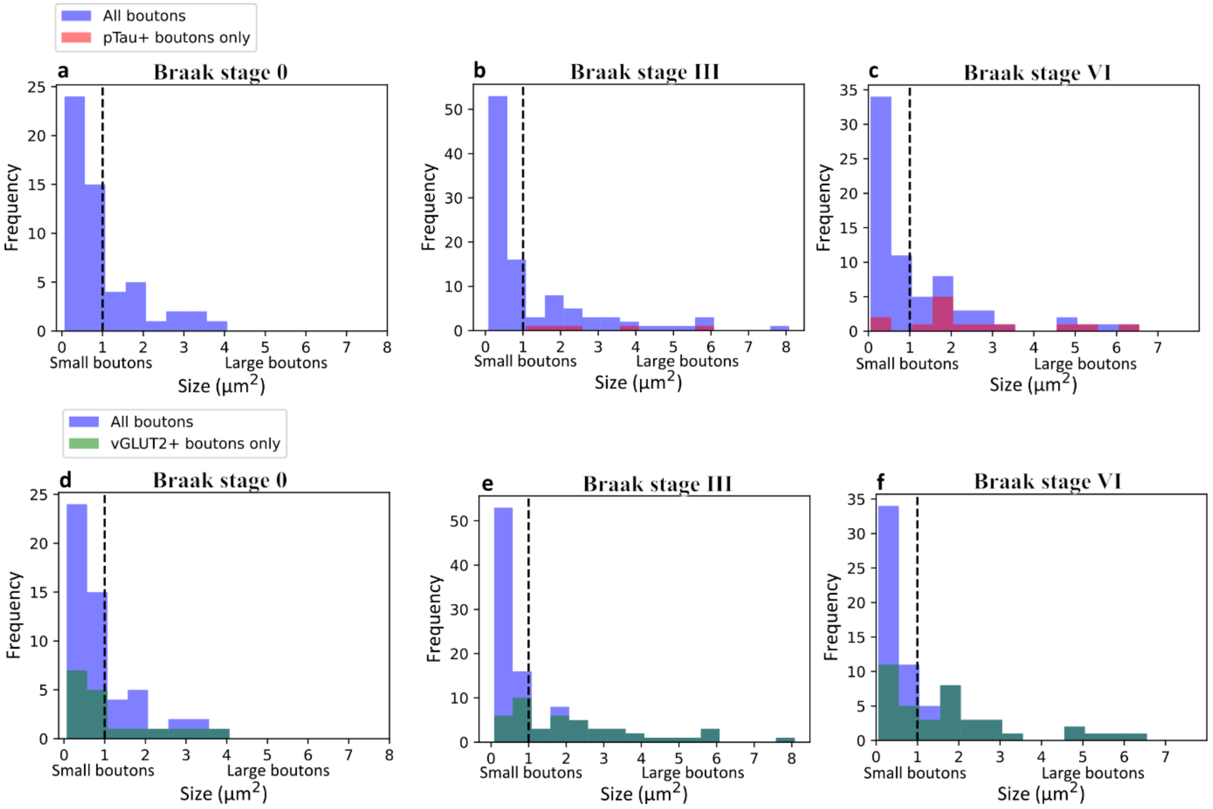

Figure S6. Distribution of the sizes of synaptic boutons in the ADn.
Histograms for the sizes of synaptic boutons - (blue) along with the subset that contained ptau (**a-c**) vGLUT2 (**d-f**). Early stage: Case 4; middle stage: Case 12; late stage: Case 17.

Primary antibody	Concentration and dilution	Epitope	Specificity information	Source	Identifier
Mouse anti-AT8	200 µg/mL, 1:2500 for DAB 1:5000 for IF	Phosphorylation at S202 and T205 residues of human Tau	Immunoblot. AT8 did not recognise wildtype or mutated recombinant tau proteins prior to brain extract phosphorylation (Goedert et al., 1995)	Thermo Fisher Scientific	Cat# MN1020; RRID: AB_223647
Rabbit anti-Calretinin	Antiserum, 1:1000 for DAB 1:2000 for IF	Polyclonal, recombinant human calretinin with 6-his tag	Western blot supplied by Swant. No signal in knockout animals	Swant	Cat# 7699/3H; RRID: AB_10000321
Mouse anti-vGLUT2	Purified, 1:500 for DAB	Recombinant rat vGLUT2 amino acids 510-582	Similar to immunoreactivity characterized in mouse hippocampus by Herzog et al. 2006 J. Neurochem.	Sigma Aldrich	Cat# MAB5504; RRID: AB_2187552
Guinea pig anti-vGLUT2	Antiserum, 1:8000 for DAB with Tyramide	Recombinant protein from rat vGLUT2	Evaluated by Western Blot on Mouse brain lysates (Sigma Aldrich)	Synaptic Systems	Cat# 135 404; RRID: AB_887884
Mouse anti-CP13	Cell culture supernatant, 1:1000 for DAB	Detects Tau phosphorylated at S202.	Tau knock out mice	Dr Peter Davies	
Mouse anti-PHF-1	Cell culture supernatant, 1:1000 for DAB	Around S396 and S404 phosphorylated sites	Tau knock out mice	Dr Peter Davies	

Table S1. Primary antibodies.

**Chemicals**		
Normal goat serum	Vector Laboratories	Cat# S-1000
Normal horse serum	Vector Laboratories	Cat# S-2000
Cold Water Fish Skin Gelatin	Aurion	Cat# 900.033
Enhancement conditioning solution (ECS)	Aurion	Cat# 500.055
Silver enhancement solution SE-LM	Aurion	Cat# 500.022
Diaminobenzidine (DAB)	Sigma-Aldrich	Cat# D5637-1G
Chromium(III) potassium sulfate dodecahydrate	Sigma-Aldrich	Cat# 243361
Gelatin from bovine skin	Sigma-Aldrich	Cat# G9391
Acetonitrile	VWR Chemicals	Cat# 83657.32
DPX Mountant	Merck	Cat# 6522
Donkey anti-mouse Alexa Fluor 488	Thermo Fisher Scientific	Cat# A-21202
Donkey anti-rabbit Cy3	Jackson Immuno Research	Cat# 711-165-152
Biotinylated Goat anti-mouse	Vector Laboratories	Cat# BA-9200-1.5
Goat-anti-Mouse IgG (H&L) Ultra Small	Aurion	Cat# 800.022
**Critical commercial assays**		
Vectastain ABC Elite kit	Vector Laboratories	Cat# PK6100; RRID: AB_2336819
Vectashield Antifade Mounting Medium	Vector Laboratories	Cat# H-1000; RRID: AB_2336789
TSA Biotin Reagent Pack	Akoya Biosciences	Cat# SAT700001EA
**Software and algorithms**		
Fiji	ImageJ	https://imagej.net/software/fiji
Zen 2008, Zen Black, Zen Blue,	Zeiss	www.zeiss.co.uk
Python		https://www.python.org/
CaseViewer	3DHISTECH	https://www.3dhistech.com/solutions/caseviewer
QuPath	Bankhead et al.	https://qupath.github.io/
TrakEM2	Cardona et al.	https://www.ini.uzh.ch/~acardona/trakem2.html

Table S2. Other reagents and resources.

Location Stage:	0	I	II	III	IV	V	VI
ADn	0(1), 1(2), 2(1)	1(2)	1(1), 1.5(1), 2(1)	1(1), 2(3)	2(1)	3(2)	3(1)
AV	0(4)	0(3)	0(4)	0 (3), 1(1)	1(1)	2(1)	2(1)
LD	NA	0(1)	0(1)	0(1), 1(1)	NA	3(1)	2(1), 3(1)
MD	0(4)	0(3)	0(3)	0 (3) 1(1)	0(1)	1(1)	1(2)
PVT	0(2), 0.5(2)	0(2)	0(2), 1(0.5)	0(1), 0.5(1), 1(1), 2(1)	2(1)	2(1), 3(1)	2(1), 3(1)
TRN*	0(2), 0.5(3)	0(2)	0(1), 0.5(3)	0(1), 1(4)	0(1)	2(2)	2(2)
RE	0(2), 0.5(1)	0(2)	0(1), 0.5(2)	0(1), 1(3)	1(1)	2(1)	2(2)
DG	0(3)	NA	NA	0(1), 0.5(1), 1(1)	NA	NA	2(1), 3(1)
CA3	0(1), 0.5(1), 1(1)	NA	NA	1(1), 2(1), 3(1)	NA	NA	3(2)
CA2	0(1), 0.5(1), 1(1)	NA	NA	1(1), 2(1), 3(1)	NA	NA	3(2)
CA1	0(2), 1(1)	NA	NA	2(1), 3(2)	NA	NA	3(2)
Prosubiculum	0(2), 2 (1)	NA	NA	2(2), 3(3)	NA	NA	3(2)
Subiculum	0(2), 1(1)	NA	NA	1(2), 2(1)	NA	NA	3(2)
Presubiculum	0(2), 0.5(1)	NA	NA	0(1), 1(2)	NA	NA	3(2)
Parasubiculum	0(2), 1(1)	NA	NA	1(2), 3(1)	NA	NA	3(2)
Entorhinal area	0(1), 1(1), 2(1)	NA	NA	3(3)	NA	NA	3(1)
RS (BA30, BA 29, BA 26)*	0(1), 1(1)	NA	NA	3(3)	NA	NA	3(2)
BA 23	0(2)	NA	NA	1(3)	NA	NA	3(1), 2(1)

Table S3. Intensity scoring for ptau in the rostral thalamus and cerebral cortex.
The distribution of ptau in each area was defined by the following median scores: 0, lacking detectable ptau; 0.5, containing trace inclusions; 1, sparse; 2, moderate; 3, dense. Numbers in parentheses show the number of cases with that score out of the total in the given stage. *Axons containing ptau were sometimes present even when score 0 was given. For the RS, at early and middle stages, ptau was concentrated in BA 26 and 29, with one case (Case 12) exhibiting some ptau in BA 30. NA, not available.

**Case**	**Braak stage**	**Thalamic nucleus**	**Ptau coverage (%)**	**Ptau cell frequency (cell/mm^2)**
**4**	0	AV	0.07	0
		ADn	0.84	2.58
		MD	0.06	0
		PVT	0.66	0.32
		TRN	0.08	0
**5**	0	AV	0.09	0
		ADn	0.28	0
		MD	0.05	0
		PVT	0.11	0.04
		TRN	0.07	0
**11**	III	AV	0.08	0.11
		ADn	3.31	4.97
		MD	0.07	0.02
		PVT	0.13	0.05
		TRN	0.04	0
**12**	III	AV	0.17	0.03
		ADn	12.14	15.13
		MD	0.19	0
		PVT	0.4	0.47
		TRN	0.09	0
**13**	III	AV	0.31	0.32
		ADn	4.52	14.84
		MD	0.3	0.93
		PVT	1.99	5.2
		TRN	0.19	0.03
**14**	IV	AV	0.18	0.14
		ADn	14.2	19.59
		MD	-	-
		PVT	1.34	4.58
		TRN	0.07	0
**17**	VI	AV	6.28	14.49
		ADn	36.31	0
		MD	1.62	2.84
		PVT	11.62	29.13
		TRN	2.98	0

Table S4. Quantification of ptau coverage and immunopositive cell counts.
Pixel-classifier quantification of ptau coverage and cell counts from perfusion-fixed tissue.
